# Genome-wide identification and multi-dimensional functional characterization of the SIR2 family in *Brassica napus* L.

**DOI:** 10.1371/journal.pone.0340688

**Published:** 2026-01-22

**Authors:** Hanbing Hu, Ling He, Rui Chen, Ziheng Jing, Heping Wan, Zilan Xiao, Hongtao Cheng, Chunhong Wu, Xiaoyun Liu

**Affiliations:** 1 Hubei Engineering Research Center for Protection and Utilization of Special Biological Resources in the Hanjiang River Basin, College of Life Science, Jianghan University, Wuhan, China; 2 Key Laboratory of Biology and Genetic Improvement of Oil Crops, Oil Crops Research Institute of Chinese Academy of Agricultural Sciences, Wuhan, China; China Three Gorges University, CHINA

## Abstract

Silent Information Regulator 2 (SIR2) family proteins are NAD^+^-dependent histone deacetylases that play key roles in epigenetic regulation, plant development, and stress responses; however, systematic characterization of this family (hereafter SRTs) in the important oilseed crop *Brassica napus* (rapeseed) remains limited. In this study, we identified 4 SRT genes in the rapeseed genome, designated BnaASRT1/2 and BnaCSRT1/2, based on their homology to Arabidopsis SRTs and chromosomal localization. These genes encode proteins with conserved SIR2 domains, sharing >80% sequence identity and >95% coverage with Arabidopsis SRTs, and exhibit similar subcellular localizations (SRT1s in the nucleus, SRT2s in mitochondria). Phylogenetic and synteny analyses confirmed the evolutionary conservation of rapeseed SRTs, which originated from whole-genome duplication events and were stably inherited from its diploid ancestors (*Brassica rapa* and *Brassica oleracea*). Structural analysis indicates that gene structure, motifs, and promoter elements related to stress and hormone responses are conserved, although subtle differences in the promoters suggest functional specialization. Notably, GO enrichment and molecular docking assays demonstrated that BnaSRTs possess histone deacetylase activity, with strong binding affinity to key acetylation sites (e.g., H3K9ac). Treatment with the SIRT-specific inhibitor nicotinamide (NAM) significantly elevated histone acetylation levels and altered the expression of genes involved in photosynthesis, metabolism, and stress responses-further supporting BnaSRTs' ole in epigenetic regulation. Furthermore, expression profiling via the BnIR database showed tissue-specific expression of *BnaSRTs* (e.g., high *BnaASRT2* expression in buds, flowers, and siliques) and dynamic responses to abiotic stresses (salt, drought, cold) and hormones (ABA, JA, IAA), with *BnaSRT2s* showing the most prominent expression changes. Collectively, these findings clarify the evolutionary conservation, structural features, and functional roles of the SIR2 family in rapeseed, laying a foundation for epigenetic improvement of its agronomic traits.

## Introduction

Plants have evolved sophisticated epigenetic regulatory mechanisms to maintain stable growth and development under diverse environmental conditions [1]. Histone modification represents one of the key epigenetic regulatory strategies employed by plants in response to environmental stresses [2,3].

Histone acetylation, among the most common histone modifications, is catalyzed by histone acetyltransferases (HATs) and histone deacetylases (HDACs), with HATs mediating acetylation and HDACs governing deacetylation [4]. The main function of HDACs is to remove acetyl groups from Nε-Acetyl-L-lysine residues on histones, thereby increasing the number of positive charges on the histones and enhancing their ability to bind DNA [5]. This process enhances the accessibility of chromatin to transcriptional regulators, thereby promoting gene expression. In contrast, the deacetylation of histones by HDACs leads to chromatin compaction, thereby inhibiting gene expression [6]. Three HDAC families have been identified in plants: Class I, designated as the Reduced Potassium Dependency 3 (RPD3)/Histone Deacetylase 1 (HDA1) family; Class II, the Silent Information Regulator 2 (Sirtuin/SIR2) family; and Class III, the HD2/HDAC tuin (HD-tuin/HDT) family [7,8]. The SIR2 gene family is a group of highly conserved NAD ⁺ -dependent histone deacetylases in eukaryotes, with vital effects on plant growth, development, and their responses to stress conditions. [9,10].

In plants, extensive research has been documented regarding the involvement of the SIR2 gene family in the regulation of energy metabolism and hormone signaling pathways, and these two processes further adjust plant growth and development along with responses to abiotic and biotic stresses. Through histone deacetylation, AtSRT1 directly suppresses the expression of *FT* and *SOC1*. Simultaneously, it directly downregulates *TOR* (a central hub of glucose-driven energy signal transduction), thereby promoting the deposition of the H3K27me3 marks on *FLC* through the TOR-FIE-PRC2 pathway, ultimately enhancing the suppression of flowering [9]. In rice, OsSRT1 inhibits glycolysis by deacetylating OsGAPDH, increases glucose content in rice plants, and regulates starch accumulation in seeds [11]. Furthermore, OsSRT1 negatively regulates leaf senescence by inhibiting the expression of biosynthetic genes involved in the MeOH-jasmonates cascade metabolic pathway [12]. AtSRT2 exhibits a primary localization in mitochondria, where it exerts a crucial effect on the fine-tuning of energy metabolism within this organelle [13]. Disruption of AtSRT2 results in decreased ATP content while causing elevated ADP levels in the leaves of *Arabidopsis* [14]. AtSRT1 and AtSRT2 interact with EIN2 NUCLEAR ASSOCIATED PROTEIN1 (ENAP1), and this protein complex exerts a mediating role in transcriptional repression via the regulation of H3K9 acetylation levels within the ethylene signaling transduction pathway [15]. PbSRT1 and PbSRT2 play important roles in regulating pear fruit growth and ripening by modulating hormone and sugar metabolism [16]. FaSRT2-1 plays a significant role in regulating energy metabolism, promoting the maturation, aging, and quality formation of strawberry fruits [17].

In *Arabidopsis*, AtSRT1 negatively controls the stress tolerance of plants as well as the glycolytic process; concurrently, it stimulates mitochondrial respiration by means of epigenetic regulation and adjusting the transcriptional activity of AtMBP-1 [18]. AtSRT2 promotes plant growth and represses osmotic stress-associated signaling [10]. Additionally, AtSRT2 may negatively regulate basal resistance against the pathogen *Pseudomonas syringae* pv. tomato DC3000 (Pst DC3000) and the expression of pathogenesis-related gene 1 (*PR1*) by inhibiting salicylic acid (SA) biosynthesis [19]. Under salt treatment, AtSRT2 modulates histone acetylation in the promoter region of *Vesicle-associated membrane protein 714* (*VAMP714*) and represses its transcription, thereby facilitating the trafficking of H₂O₂-containing vesicles in cells and enhancing salt tolerance [20]. In *Oryza sativa* (rice), RNA interference (RNAi)-mediated silencing of OsSRT1 induces increased histone H3K9 acetylation and decreased H3K9 dimethylation, which in turn triggers H₂O₂ production, DNA fragmentation, cell death, and hypersensitivity reactions. By contrast, *OsSRT1* overexpression enhances oxidative stress tolerance [21]. In strawberry, FaSRT1-2 positively promotes vegetative growth and fruit ripening by regulating the ABA and GA pathways, while negatively modulating resistance to heat stress and *Botrytis cinerea* infection via controlling the expression of relevant genes (e.g., heat shock proteins, transcription factors and stress-related genes) [22]. DgSRT2 enhances cold tolerance in chrysanthemum via the reactive oxygen species (ROS) scavenging system [23]. Recently, significant breakthroughs have been made in functional studies of TaSRT1 in wheat. Research indicates that TaSRT1 directly interacts with the key drought resistance factor TaDT-A, regulating the protein stability and transcriptional activity of TaDT-A through lysine deacetylation, thereby influencing the drought resistance of wheat [24]. Furthermore, TaSRT1 negatively regulates *Fusarium* crown rot (FCR) resistance by inhibiting the accumulation of TaHSP18.6 via deacetylation. TaHSP18.6 interacts with TaIAA1 to regulate resistance by altering endogenous auxin content, thus proposing a TaSRT1-TaHSP18.6 regulatory model for wheat FCR resistance [25].

*Brassica napus* L. (rapeseed), the second largest oilseed crop worldwide, possesses an allotetraploid genome. Extensive research indicates that the SIR2 family plays a crucial role in plant growth, development, and stress responses. However, the SIR2 family in rapeseed remains inadequately characterized. Thus, it is essential to elucidate their functional properties in this species.

In the present study, four SIR2 family members were identified in the rapeseed genome, followed by comprehensive analyses encompassing phylogeny, conserved domains, gene structures, and cis-acting elements. Furthermore, the biochemical functions of BnaSRTs were predicted via molecular docking and validated using sirtuin inhibitor. Their expression profiles were also investigated across different developmental stages of various rapeseed organs, as well as in responses to abiotic stresses and hormone treatments. In summary, this study conducted a systematic analysis of the *SRTs* in rapeseed, laying a solid foundation for deciphering their regulatory roles in rapeseed growth and development, stress responses, and hormonal signal transduction.

## Materials and methods

### Characterization and Categorization of *SRTs*

Following previous research, the SRTs protein sequences of *Arabidopsis thaliana* and *Oryza sativa* were downloaded from the Plant Comparative Genomics portal (Phytozome, https://phytozome-next.jgi.doe.gov/) [26] and the sequences of *Homo sapiens* were downloaded from the National Center for Biotechnology Information (NCBI, https://www.ncbi.nlm.nih.gov/) [27]. The *Arabidopsis* SRTs protein sequences were used as queries for BLAST analysis on the *Brassica napus* multi-omics information resource (BnIR, https://yanglab.hzau.edu.cn) [28], to preliminarily identify *SRTs* in the *Brassica napus* Zhongshuang11. Gene IDs are available in Supplementary S1 Table. Conserved protein domains of the target gene family in *Brassica napus* were analyzed via the NCBI Conserved Domain Database (CDD, https://www.ncbi.nlm.nih.gov/Structure/cdd/wrpsb.cgi) [29].

Physicochemical properties of proteins in the target gene family were analyzed via the ExPASy online software ProtParam (https://web.expasy.org/protparam/) [30], with parameters encompassing amino acid count, theoretical pI, molecular weight, instability index, aliphatic index, and grand average of hydropathicity. The CDS length, number of introns, number of exon and chromosome location of each confirmed *SRTs* was retrieved from the BnIR. Amino acid sequences of two AtSRTs and four BnaSRTs were used to predict the sub-cellular localization for plant proteins by ProtComp 9.0 on the Softberry website. (http://www.softberry.com/berry.phtml?topic=protcomppl&group=programs&subgroup=proloc).

### Protein structures prediction

The structure of the BnaSRTs proteins were predicted and modeled using the SWISS-MODEL online software (https://swissmodel.expasy.org/interactive) [31], and then visualized using PyMOL 2.6 (https://pymol.org/) [32]. Two AtSRTs and four BnaSRTs protein sequences were simultaneously aligned with Clustal Omega (https://www.ebi.ac.uk/jdispatcher/msa/clustalo) [33], and labeled as SIR2 domains, that can be found in S1 Fig. The secondary structure was visualized in PyMOL 2.6, consisting of three structures: Helix, Sheet and Loop, and conserved domains are also displayed based on the results of the analysis on the NCBI’s CDD.

### Phylogenetic analysis of SRTs proteins in four species

The identified *Brassica napus* SRTs protein sequences and those of three other species, including *Homo sapiens*, *Arabidopsis thaliana* and *Oryza sativa*, were downloaded, and the protein sequences were simultaneously aligned with MUSCLE. The phylogenetic tree was constructed using the Molecular Evolutionary Genetics Analysis 11 (MEGA11, https://www.megasoftware.net/) [34] by the neighbor-joining (NJ) method, and a bootstrap analysis was conducted using 1000 replicates at each node. The phylogenetic tree was visualized and optimized by Interactive Tree Of Life (iTOL, https://itol.embl.de/) [35]. Different colors were used to indicate the homologous genes of two AtSRTs, and different shapes were used to label the SRTs proteins of different species.

### Chromosomal distribution and synteny analysis

The genomic data of *Arabidopsis thaliana*, *Brassica napus, Brassica rapa* and *Brassica oleracea* were obtained from the *Arabidopsis* Information Resource (TAIR, https://www.arabidopsis.org/) [36] and BnIR, respectively. The gene location analysis and gene density analysis were performed using the Gene Location Visualize form GTF/GFF tool and Gene Density Profile tool in TBtools-Ⅱ v2.149 (https://github.com/CJ-Chen/TBtools) [37]. The synteny analysis was performed using the One Step MCScanX tool, Dual Systeny Plot tool, File Merge For MCScanX tool and Multiple Synteny Plot tool in TBtools-Ⅱ.

### Sequence analysis

The genomic data of *Homo sapiens* and *Oryza sativa* were obtained from the GenBank in NCBI (https://www.ncbi.nlm.nih.gov/genbank/) [38] and Rice Genome Annotation Project (RGAP, https://rice.uga.edu/) [39], respectively. The conserved protein domain SIR2 was searched within protein sequences of SRTs using the NCBI’s CDD. The gene structure of *SRTs* were assessed utilizing information from tree plant genome GFF3 file. And the motif consensus can be found in the S2 Table. The conserved features of SRTs protein sequences of four species were analyzed using the Multiple Em for Motif Elicitation v5.5.8 (MEME Suite, https://meme-suite.org/meme/tools/meme) [40]. The domains, gene structures and motifs were visualized using the Gene Structure View (Advanced) tool in TBtools-Ⅱ. The cis-elements in the promoters of four *Brassica napus SRTs* were identified using the PlantCare (https://bioinformatics.psb.ugent.be/webtools/plantcare/html/) [41], and visualized using the Gtf/Gff3 Sequences Extractor tool, the Fasta Stater tool, the Fasta Sequence Manipulator tool and the Simple BioSequence Viewer tool in TBtools-Ⅱ.

### GO enrichment analysis and molecular docking prediction

Based on the protein sequences of BnaSRTs, protein GO enrichment analysis was performed using STRING online software (https://cn.string-db.org/) [42]. Due to the lack of relevant information on the ZS11 rapeseed gene in the STRING, information on the Darmor rapeseed gene, which is homologous to the ZS11 rapeseed gene, was used. Details of GO and KEGG pathway enrichment analysis can be found in S3 Table.

5 types of ligand-modified histone acetylation sites (H3K9ac, H3K14ac, H3K23ac, H3K27ac and H4K5ac) were downloaded from the RCSB Protein Data Bank (RCSB PDB, https://www.rcsb.org/) [43]. Receptor and ligand proteins were subjected to operations such as dehydration, hydrogenation, and energy minimization using AutoDock4 (https://autodock.scripps.edu/) [44], and molecular docking was performed using AutoDock Vina (https://vina.scripps.edu/) [45] to calculate the results. The results were visualized using PyMOL and the online software PoseEdit (https://proteins.plus/) [46].

### Treatment of rapeseed with histone deacetylase inhibitors, RNA-seq and identification of histone modification levels

The ZS11 rapeseed seeds were germinated by soaking in clean water, and after 3 days, they were transferred to a modified Hogue’s nutrient solution for rapeseed cultivation in a greenhouse. Inside the greenhouse, a constant temperature of 20°C, constant humidity, 20 hours of illumination, and 4 hours of darkness. After 7 days, a portion of the rapeseed was used as a control group, while another portion was treated with Nicotinamide, one of the histone deacetylase inhibitors (with a working concentration of 50μM) [11]. On the fifth day post-treatment, significant phenotypic changes were observed. These samples are first cleaned with ultra-pure water and then preserved in liquid nitrogen.

Total RNA was extracted using TRIzol reagent (Invitrogen Life Technologies). The RNA was subsequently evaluated and quantified in two steps: 1) its purity and concentration were measured using a NanoDrop spectrophotometer (Thermo Fisher Scientific) and a Qubit 4.0 fluorometer (Thermo Fisher Scientific); 2) the integrity and quantity of RNA were assessed using the Agilent 2100/4200 system. Three micrograms of RNA were used as the starting material for subsequent sample preparation. cDNA libraries were constructed through Poly(A⁺) enrichment and using Hieff NGS® DNA Selection Beads. After library construction, the concentration was measured again with Qubit 4.0. The libraries were then sent to 563 Sci‑Service Biotechnology Co., Ltd. (Wuhan, China) for sequencing on the DNBSEQ‑T7 platform using the PE150 mode. The RNA‑seq experiment was conducted with three biological replicates. The raw FASTQ data were first subjected to quality control using fastp (v0.21.0), filtering out low-quality sequences to obtain high-quality clean reads. The clean reads were then aligned to the reference genome using HISAT2 (v2.1.0). Gene read counts were quantified using StringTie (v2.1.5), and expression levels were normalized as FPKM (fragments per kilobase of transcript per million mapped reads). Differentially expressed genes (DEGs) were identified using DESeq2 (v1.34.1) with the criteria of |log₂FoldChange| > 1 and Padj ≤ 0.05. Finally, the identified DEGs were submitted to the GO and KEGG databases for functional annotation (Padj ≤ 0.05) and their functions as well as metabolic pathways were analyzed and predicted. GO and KEGG pathway enrichment analyses for differentially expressed genes, histone‑modifying genes, and genes whose expression is regulated by these modifications were carried out using AgriGOv2. (http://systemsbiology.cau.edu.cn/agriGOv2/) [47] and KOBAS software [48] (S7 Table).

Additionally, total proteins extracted from the samples were used for Western Blotting to detect the changes in the levels of five histone modifications: H3K9ac (A7255, ABclonal), H3K14ac (A7254, ABclonal), H3K23ac (A18154, ABclonal), H3K27ac (A22264, ABclonal), and H4K5ac (A23080, ABclonal), in order to identify the biochemical functions of SRTs in rapeseed.

### Expression profiling of *SRTs* in rapeseed

The expression pattern of *SRTs* in rapeseed following tissue, abiotic stress treatment and hormone stress treatment were obtained from BnIR. The study types were as follows: (1) tissues of leaf and root: bud, filament, petal,pollen, sepal, cotyledon, vegetative rosette, leaf, root, seed, silique, and so on. For detailed information, please refer to S4 Table. (2) abiotic stress treatment of leaf and root: salt, drought, freezing, cold, heat, and osmotic. For detailed information, please refer to S5 Table. (3) hormone treatment of leaf and root: Indole-3-acetic acid (IAA), 1-Aminocyclopropane-1-carboxylic Acid (ACC), Gibberellin (GA), Abscisic acid (ABA), Trans-Zeatin-Riboside (TZ), Jasmonic acid (JA), and Brassinolide (BL). For detailed information, please refer to S6 Table. RNA-seq data was downloaded from the BnIR, with the gene expression quantification method based on Kallisto, and gene expression was estimated using the transcripts per kilobase of exon model per million mapped reads (TPM) method.

### Validation of transcriptome results and expression patterns via qRT-PCR

Total RNA was extracted from rapeseed materials treated with NAM (50 μM NAM, 5 days), salt stress (1.3% NaCl, 2 days), and drought stress (20% PEG6000, 1 day) using Trizol reagent. From the extracted total RNA, 1 µg was reverse‑transcribed into cDNA using the ABScript II First‑Strand cDNA Synthesis Kit (RK20400, ABclonal). The resulting first‑strand cDNA was then used as the template for the subsequent qRT‑PCR assays.

qRT‑PCR was performed on an ABI 7900 instrument. The cycling protocol began with an initial denaturation at 95°C for 15 seconds, followed by 42 amplification cycles consisting of 95°C for 5 seconds and 60°C for 40 seconds.

For melt‑curve analysis, the program included a step at 95°C for 15 seconds, then 60°C for 20 seconds, and a final step at 95°C for 15 seconds. Data acquisition and analysis were carried out using the ABI 7900 system. Relative gene expression was calculated with the 2^⁻ΔΔCT^ method, and BnActin7 (EV116054) from rapeseed was used as a reference gene. The primers employed in these analyses are presented in S8 Table.

## Results

### Identification of *SRTs* in rapeseed

To identify the *SRT* genes in the rapeseed (*Brassica napus* L.) genome, we performed a comprehensive search using *Arabidopsis* SRTs sequences as queries. The results revealed four *SRT* genes harboring the SIR2 domain in the rapeseed genome (Table 1). Based on their chromosomal locations and homology to *Arabidopsis SRTs*, these genes were designated as *BnaASRT1/2* and *BnaCSRT1/2*, respectively. Physicochemical properties of the identified genes were analyzed via the ProtParam server. Our analyses showed that the lengths of rapeseed SRTs proteins ranged from 357 to 484 amino acids (aa); their molecular weights varied between 39.63 and 54.20 kDa; and their isoelectric points (pI) spanned from 6.62 to 8.96. Specifically, BnaASRT1 and BnaCSRT1 were weakly acidic proteins with pI > 6, whereas BnaASRT2 and BnaCSRT2 were basic proteins with pI > 8. The instability index ranged from 42.3 to 52.25; the aliphatic index was between 78.46 and 87.03; and the grand average of hydropathicity (GRAVY) varied from −0.246 to −0.316, indicating that BnaSRTs proteins exhibit strong hydrophilicity (Table 1). Prediction results suggested that the corresponding *SRTs* genes on the A and C subgenomes of rapeseed share identical subcellular localization and display similarities in isoelectric point, aliphatic index, and GRAVY. BnaASRT1, BnaCSRT1, and AtSRT1 were all localized in the nucleus, while BnaASRT2, BnaCSRT2, and AtSRT2 were all targeted to mitochondria (Table 1). Additionally, the SRTs of rapeseed and *Arabidopsis* showed similarities in molecular weight, instability index, aliphatic index, and GRAVY (Table 1).

**Table 1:** Physicochemical properties of *SIR2* gene family members in *Arabidopsis thaliana* and *Brassica napus*. No.aa, Number of Amino Acid; TPI, Theoretical pI; MW, Molecular Weight; II, Instability Index; AI, Aliphatic Index; GRAVY, Grand Average of Hydropathicity; PSL, Prediction of Subcellular Localisation; N, Nuclear; M, Mitochondrial.

Gene Name	Gene ID	Species	No. aa	TPI	MW (kDa)	II	AI	G*RAV*Y	PSL
*AtSRT1*	*AT5G55760*	*Arabidopsis thaliana*	473	8.37	52.64	45.37	90.44	-0.236	N
*BnaASRT1*	*BnaA03G0119500ZS*	*Brassica napus*	484	6.62	54.20	52.25	84.13	-0.246	N
*BnaCSRT1*	*BnaC03G0138400ZS*	*Brassica napus*	468	6.87	52.38	48.01	87.03	-0.267	N
*AtSRT2*	*AT5G09230*	*Arabidopsis thaliana*	376	9.08	41.87	46.26	76.04	-0.31	M
*BnaASRT2*	*BnaA02G0030900ZS*	*Brassica napus*	357	8.96	39.69	42.3	79.83	-0.291	M
*BnaCSRT2*	*BnaC02G0034200ZS*	*Brassica napus*	357	8.81	39.63	43.45	78.46	-0.316	M

**Fig 1:**
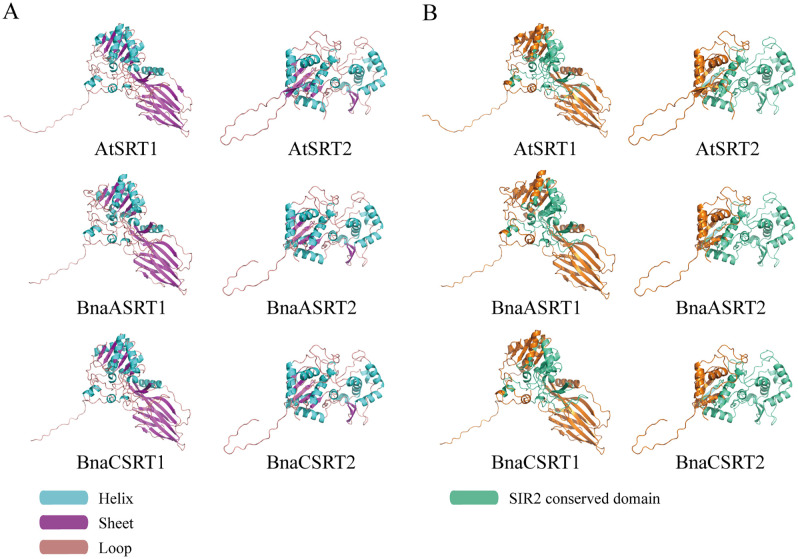
Protein structures prediction. **(A)** The secondary structure of the protein is shown within the tertiary structure. The secondary structures are distinguished by blue helices, purple sheets, and pink loops. **(B)** In the tertiary structure of the protein, the SIR2 conserved domain is displayed in green.

Protein sequence alignments were conducted between AtSRT1 and BnaASRT1/BnaCSRT1, as well as between AtSRT2 and BnaASRT2/BnaCSRT2, respectively. The alignment results demonstrated that the coverage (cov) of SRT proteins between rapeseed and *Arabidopsis* exceeded 95%, with a percent identity (pid) of over 80%, indicating that rapeseed and *Arabidopsis* SRTs share a common evolutionary origin and maintain a close sequence relationship (S1 Fig). AlphaFold was used to predict the tertiary structures of BnaSRTs proteins (Fig 1), and their secondary structural elements—including helices, sheets, and loops—were visualized using PyMOL (Fig 1A). Furthermore, the positions of the SIR2 domains, as predicted by CDD, were mapped onto the tertiary structures of BnaSRTs (Fig 1B). Structural predictions revealed a high degree of similarity in both secondary and tertiary structures between AtSRT1 and BnaASRT1/BnaCSRT1, as well as between AtSRT2 and BnaASRT2/BnaCSRT2. These findings suggest that SRT proteins are highly conserved between *Brassica napus* and *Arabidopsis*, two species belonging to the *Brassicaceae* family.

### Chromosomal distribution and phylogenetic relationship analysis of BnaSRTs genes

**Fig 2:**
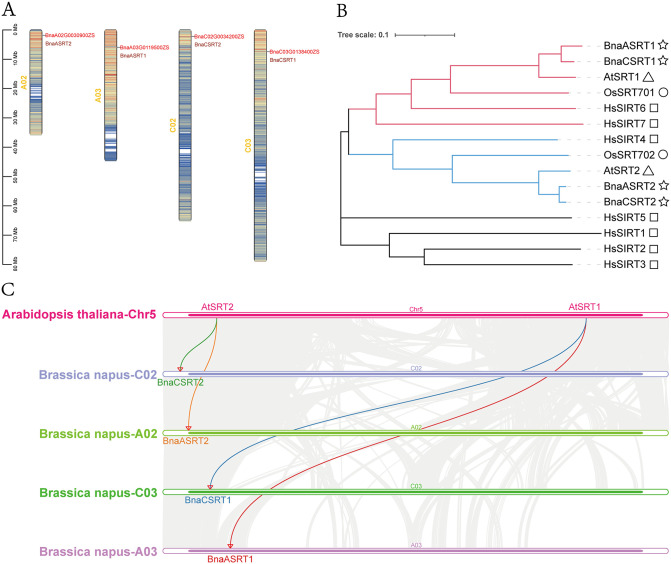
Chromosomal Distribution, Synteny Analysis of *SRTs* Genes, and Phylogenetic Analysis of *SRTs.* **(A)** Chromosomal distribution of *BnaSRTs* in rapeseed. The gene density heat map within the chromosome rectangles indicates gene density from low (cool colors) to high (warm colors). **(B)** Phylogenetic analysis of 15 SRT proteins based on *Homo sapiens* (7), *Arabidopsis thaliana* (2), *Brassica napus* (4), and *Oryza sativa* (2). A neighbor-joining phylogenetic tree was constructed, and bootstrap consensus was derived from 1000 replicates. The red branches represent proteins that are evolutionarily related to *AtSRT1*, while the blue branches represent proteins that are evolutionarily related to *AtSRT2*. Each shape represents a distinct species. The rect is *Homo sapiens*; the triangle is *Arabidopsis thaliana*; the star is *Brassica napus*; the circle is *Oryza sativa*. **(C)** Synteny analysis of *SRTs* of *Arabidopsis thaliana* and *Brassica napus*. **(D)** Comparative synteny of *SRT* genes among *Brassica napus*, *Brassica rapa* and *Brassica oleracea*. Background gray connections indicate whole-genome duplication events, while the collinearity of the four colors is highlighted, specifically tracking the conservative *SRTs* gene pairs.

*BnaASRT1* and *BnaCSRT1* are localized on chromosomes A03 and C03 of *Brassica napus*, respectively, while *BnaASRT2* and *BnaCSRT2* are situated on chromosomes A02 and C02, respectively. All these genes are positioned at the distal ends of their respective chromosomes (Fig 2A).

To elucidate the evolutionary relationships within the *SRT* genes, a neighbor-joining phylogenetic tree was constructed based on multiple sequence alignments of 15 SRT proteins from *Homo sapiens* (7), *Arabidopsis thaliana* (2), *Brassica napus* (4), and *Oryza sativa* (2) (Fig 2B). The results revealed that *AtSRT1*, *BnaASRT1*, *BnaCSRT1*, and *OsSRT701* share a close evolutionary relationship with *HsSIRT6* and *HsSIRT7*; in contrast, *AtSRT2*, *BnaASRT2*, *BnaCSRT2*, and *OsSRT702* are evolutionarily closely related to *HsSIRT4* (S1 Table).

In order to gain a deeper understanding of the origin, evolutionary history, and functional characteristics of the *SRTs* genes in rapeseed, a synteny sequence analysis of the *SRTs* genes in *Brassica napus* and *Arabidopsis thaliana* was conducted (Fig 2C). *BnaASRT1* and *BnaCSRT1* are homologous to *AtSRT1*, while *BnaASRT2* and *BnaCSRT2* are homologous to *AtSRT2*, which is consistent with the evolutionary relationship analysis. These findings indicated that *SRTs* genes were not lost during homologous evolution and were stably inherited throughout the polyploidization process of *Brassica napus*.

To investigate the amplification mechanism of *BnaSRTs* genes in *Brassica napus* (*B. napus*), synteny analysis of *SRTs* genes was performed across the genomes of *B. napus*, *Brassica rapa* (*B. rapa*), and *Brassica oleracea* (*B. oleracea*). The analysis revealed that the four *BnaSRTs* in *B. napus* exhibit collinearity with two genes in *B. rapa* and two in *B. oleracea*, respectively, with all *BnaSRTs* mapped to chromosomes A02, A03, C02, and C03. This observation suggests that *BnaSRTs* are derived from the genomes of *B. rapa* and *B. oleracea*, and the *SRTs* gene has experienced chromosomal fragment polyploidization during its evolutionary process (Fig 2D). These results reveal a conserved intergeneric collinear relationship of *BnaSRTs*.

### Sequence and structure analysis of SRTs

**Fig 3:**
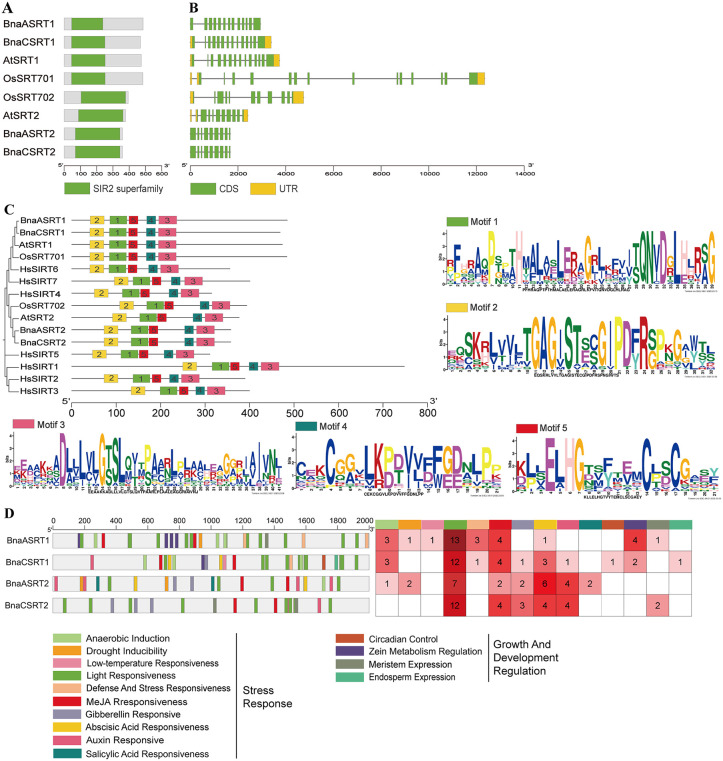
BnaSRTs features, phylogeny and cis-element analysis. **(A)** SIR2 conserved domain of BnaSRTs, AtSRTs and OsSRTs. **(B)** exon-intron structures of *BnaSRTs*, *AtSRTs* and *OsSRTs.* Green rectangles correspond to the coding sequence (CDS) or exons, yellow rectangles to untranslated regions (UTRs), and grey lines to introns. **(C)** The phylogenetic analysis and motif composition of BnaSRTs, AtSRTs, OsSRTs and HsSIRTs, and motif logo. **(D)** Analysis of Cis-Elements in *BnaSRTs* Promoters: Distribution and Quantity of Cis-Elements per Promoter.

Proteins of BnaSRTs, AtSRTs, and OsSRTs all harbor the conserved SIR2 domain (Fig 3A). Gene structure analysis revealed that *SRTs* genes from these three plant species share similar architectural features (Fig 3B). *AtSRT1*, *BnaASRT1*, *BnaCSRT1*, and *OsSRT701* exhibit identical numbers of exons and introns, with 14 exons and 13 introns, respectively. *AtSRT2* and *OsSRT702* also share consistent exon-intron counts, containing 11 exons and 10 introns. In contrast, *BnaASRT2* and *BnaCSRT2* possess 9 exons and 8 introns (Fig 3B).

The protein sequence characteristics of HsSIRTs, AtSRTs, BnaSRTs, and OsSRTs were analyzed using MEME (Fig 3C). All these SRTs proteins contain the conserved motif array 2-1-5-4-3. The motif composition of *SRT* genes from the three plant species is consistent with the predicted results of their conserved SIR2 domains (S2 Table), indicating that these SRTs proteins may encode functional homologs with similar biological functions.

Promoter elements located in the 2000-bp region upstream of the CDS of *BnaSRTs* genes were predicted using PlantCARE, and the types of promoter elements were quantified (Fig 3D). Most of these promoter elements are associated with stress responses and the regulation of plant growth and development. The promoter elements of the four *BnaSRTs* genes display substantial divergence. Although all contain light-responsive, methyl jasmonate (MeJA)-responsive, and abscisic acid (ABA)-responsive elements, their abundances vary significantly. Some promoter elements are exclusive to a single or a specific subgroup of *BnaSRT* genes: for instance, low-temperature-responsive elements are restricted to *BnaSRT1*; circadian rhythm regulation and endosperm expression elements are specific to *BnaSRT2*; defense and stress-responsive elements, as well as zein metabolism regulatory elements, are unique to *BnaSRT1*. Hormone-responsive elements—including MeJA, gibberellin, ABA, auxin, and salicylic acid responsive ones—are more abundant in *BnaSRT2* (Fig 3D).

### Analysis of BnaSRTs GO enrichment and interaction with histone acetylation

**Fig 4:**
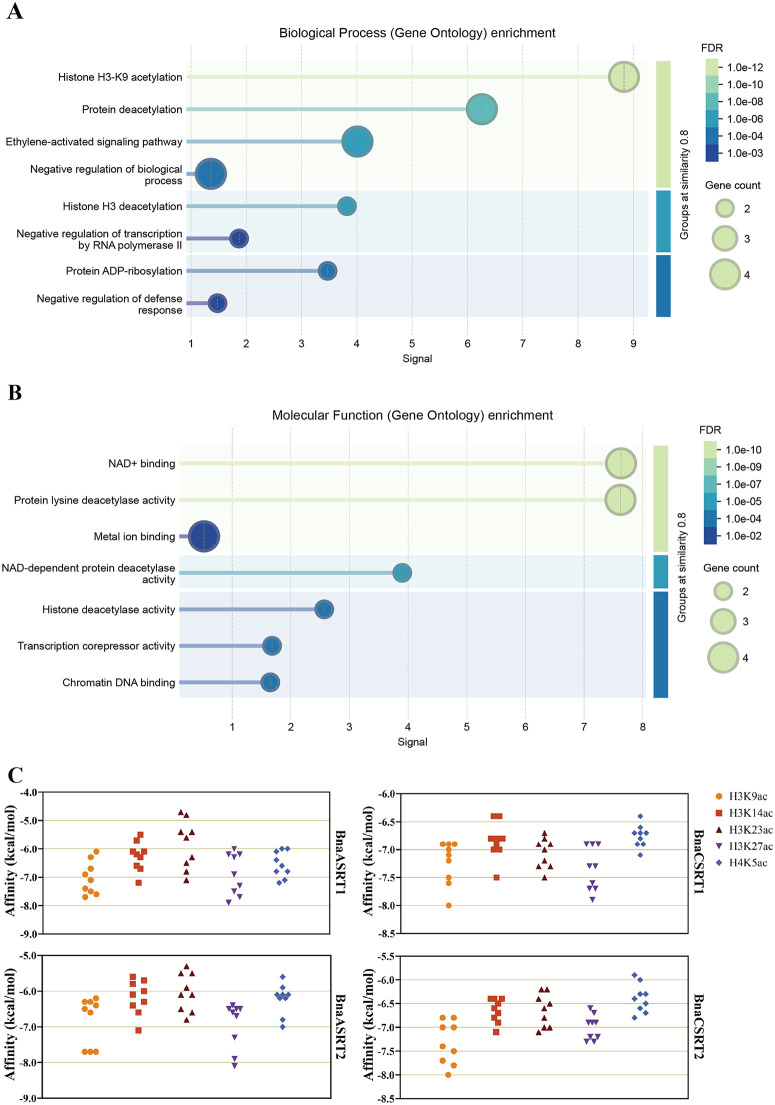
BnaSRTs GO enrichment and molecular docking to 5 histone acetylation sites. **(A)** The top 8 significantly enriched GO biological processes of BnaSRTs. **(B)** The top 7 significantly enriched GO molecular function of BnaSRTs. The horizontal axis represents the error discovery rate, while the vertical axis describes the GO terms. The size of the circles indicates the quantity of genes. **(C)** Each pair of binding models showed the affinity of 9 binding sites, and the binding pattern was analyzed with the binding site with the largest absolute affinity value. The different shapes and abscissa represent the 5 modification sites (H3K9ac, H3K14ac, H3K23ac, H3K27ac and H4K5ac), while the ordinate represents the affinity (kcal/mol).

GO enrichment analysis revealed that, in biological processes, BnaSRTs are involved not only in histone deacetylation but also in histone H3K9 acetylation, ethylene-activated signaling pathways, and negative regulation of biological processes (Fig 4A). Specifically, BnaSRT1s additionally participate in the negative regulation of RNA polymerase II-mediated transcription, while BnaSRT2s are involved in protein ADP-ribosylation and negative regulation of defense responses (S3 Table). These observations suggest that BnaSRTs may negatively regulate DNA transcription and contribute to the modulation of plant defense responses. In terms of molecular functions, BnaSRTs possess protein lysine deacetylase activity, NAD ⁺ -binding capacity, and metal ion-binding activity (Fig 4B). Notably, BnaSRT2s also exhibit chromatin DNA-binding activity (S3 Table), indicating their potential involvement in chromatin DNA modification. KEGG pathway enrichment analysis further demonstrated that BnaSRTs are implicated in nicotinic acid and nicotinamide metabolism (S2 Fig).

Molecular docking predictions showed that BnaSRTs display strong binding activity to five common histone acetylation sites (H3K9ac, H3K14ac, H3K23ac, H3K27ac, H4K5ac) (S3 Fig). Among the predicted binding sites, the highest-affinity interactions exhibit binding energies all below −6.5 kcal/mol (Fig 4C). A binding energy < −5 kJ/mol indicates a measurable binding activity between the receptor and ligand, with lower binding energies corresponding to more stable docking conformations. These results suggest that BnaSRTs are likely to interact strongly with these histone acetylation sites in vivo and may mediate deacetylation at these positions. Among the five acetylation sites, most of the four BnaSRTs show high binding capacity to H3K9ac and H3K27ac, except for BnaCSRT2, which exhibits weak binding to H3K27ac. Additionally, the binding capacity of BnaSRT2s to H4K5ac is lower than that to other H3 sites. The active pockets of BnaSRTs exhibit high diversity with diverse residue combinations; however, certain residues are highly conserved, including Glu, Thr, Ser, Asp, and Asn (S4–S7 Figs).

### Treatment of rapeseed with histone deacetylase inhibitors, differentially expressed gene analysis and biochemical functional identification

**Fig 5:**
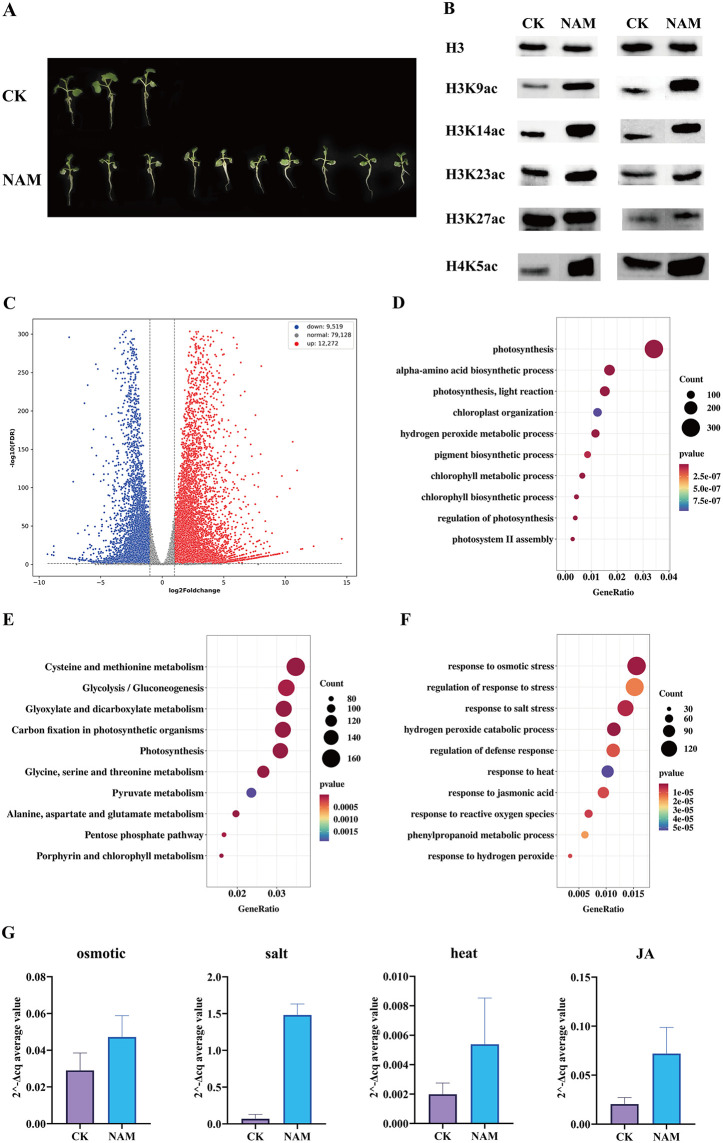
Rapeseed NAM inhibitor treatment: phenotype, biochemistry, expression and qRT-PCR validation. **(A)** Phenotype of rapeseed treated with NAM for 7 days, compared with control group (CK). **(B)** Western blotting identified the difference in histone modification levels between the inhibitor-treated group and the control group. The modified designs were H3K9ac, H3K14ac, H3K23ac, H3K27ac and H4K5ac. Each type of modification was conducted in duplicate. **(C)** Volcano plots comparing the gene expression levels for the 5-day-NAM-treated rapeseed versus untreated rapeseed. The horizontal axis represents the fold change in gene expression (log2FoldChange) between the treatment and control groups, while the vertical axis indicates the significance level of the expression difference (-log10(FDR)). Red points correspond to upregulated genes, and green points correspond to downregulated genes. Perform GO functional **(D)** and KEGG pathway **(E)** enrichment analysis on the differential gene set. 10 significant terms were selected for presentation. **(F)** In the GO functional enrichment analysis, 10 entries related to stress response were selected for presentation. The x-axis represents the ratio of the number of differential genes annotated to each GO/ KEGG term to the total number of differential genes, while the y-axis indicates the GO/ KEGG term. The size of the points represents the number of genes annotated to the GO/ KEGG term, and the color gradient from red to blue reflects the degree of significance of the enrichment. **(G)** Results of RT-PCR for NAM-treated and CK rapeseed. Four genes related to stress or hormone response functions were selected from GO functional enrichment analysis for qRT-qPCR validation. The four genes are *BnaA08G0189400ZS* (osmotic), *BnaA03G0358900ZS* (salt), *BnaA05G0333200ZS* (heat), and *BnaA06G0133300ZS* (JA). The horizontal axis represents the materials, the vertical axis represents gene expression levels (2^-ΔCq average value), and the subheadings indicate the functional type of the gene tested.

To preliminarily validate the functions of SRTs proteins in rapeseed, rapeseed plants were treated with the SIRT-specific inhibitor nicotinamide (NAM) [49] for 5 days. It was observed that the seedlings treated with NAM showed weaker growth compared to the control group (Fig 5A). Based on the assessment of histone modification levels, compared with the control (CK), NAM treatment significantly upregulated the levels of H3K9ac, H3K14ac, H3K23ac, H3K27ac, and H4K5ac modifications. This indicates a robust inhibitory effect on histone deacetylases (Fig 5B, S8 Fig), consistent with the molecular docking results.

RNA-seq analysis revealed that, relative to the control group, the inhibitor-treated group exhibited 12,272 upregulated genes and 9,519 downregulated genes (Fig 5C). GO functional enrichment analysis of the differentially expressed gene (DEG) set showed significant enrichment in biological processes (BP) such as photosynthesis, hydrogen peroxide catabolic process, cellular amino acid catabolic process, and chlorophyll metabolic process; in cellular components (CC) including photosystem, oxidoreductase complex, and photosystem I reaction center; and in molecular functions (MF) including antioxidant activity, oxidoreductase activity, flavin adenine dinucleotide (FAD) binding, and chlorophyll binding (Fig 5D). KEGG pathway enrichment analysis of DEGs demonstrated significant enrichment in pathways such as glycolysis/gluconeogenesis, glyoxylate and dicarboxylate metabolism, carbon fixation in photosynthetic organisms, photosynthesis, various amino acid metabolisms, and the pentose phosphate pathway (Fig 5E). Taken together, these findings suggest that BnaSRTs could participate in the regulation of photosynthesis and respiration. In addition, after NAM treatment, a large number of DEGs were enriched in stress response pathways, such as osmotic stress response, salt stress response, and high-temperature response (Fig 5F). From the results of GO functional enrichment analysis, we selected 4 genes with stress or hormone response functions (*BnaA08G0189400ZS* (osmotic), *BnaA03G0358900ZS* (salt), *BnaA05G0333200ZS* (heat), and *BnaA06G0133300ZS* (JA)) for qRT-PCR validation (Fig 5G). The validation results were consistent with those of the transcriptome. The above results indicate that the *BnaSRTs* gene family can regulate the stress response process of plants by influencing the level of histone deacetylation.

### Expression profiles of *BnaSRTs* in various organs, tissues and developmental stage

**Fig 6:**
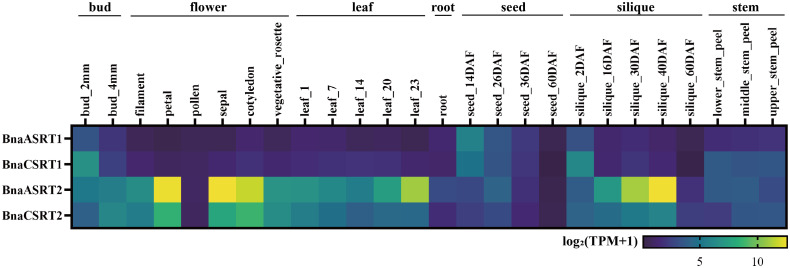
Expression profiles of the rapeseed *BnaSRTs* in various organs, tissues and developmental stages. The gradient of colors from blue to yellow represents the degree of the expression value [log_2_(TPM + 1)] provided by the color scale.

This study analyzed the expression profiles of *BnaSRTs* across diverse tissues and developmental stages of rapeseed using the publicly available transcriptome dataset from BnIR (https://yanglab.hzau.edu.cn/BnIR/) (Fig 6, S4 Table). The results demonstrated that *BnaSRTs* genes are expressed in various vegetative and reproductive organs throughout different developmental phases of rapeseed. Overall, the expression levels of *BnaASRT2* and *BnaCSRT2* in rapeseed tissues were significantly higher than those of *BnaASRT1* and *BnaCSRT1*. Specifically, *BnaCSRT1* exhibited higher expression than *BnaASRT1* in most tissues, whereas *BnaCSRT2* expression was lower than that of *BnaASRT2* in nearly all tissues examined. *BnaASRT2* and *BnaCSRT2* were predominantly expressed in buds, flowers, leaves, and siliques; in contrast, *BnaASRT1* and *BnaCSRT1* showed preferential expression in buds, seeds at stages before 34 days, and siliques within 12 days post-flowering. Notably, *BnaASRT2* displayed higher expression levels than *BnaCSRT2* in petals, sepals, and cotyledons, suggesting that *BnaASRT2* may play a more critical role in regulating flowering processes. Additionally, the expression levels of all *BnaSRT* genes in seeds exhibited a down-regulatory trend over time.

### Expression profiles of *BnaSRTs* under different abiotic stresses

**Fig 7:**
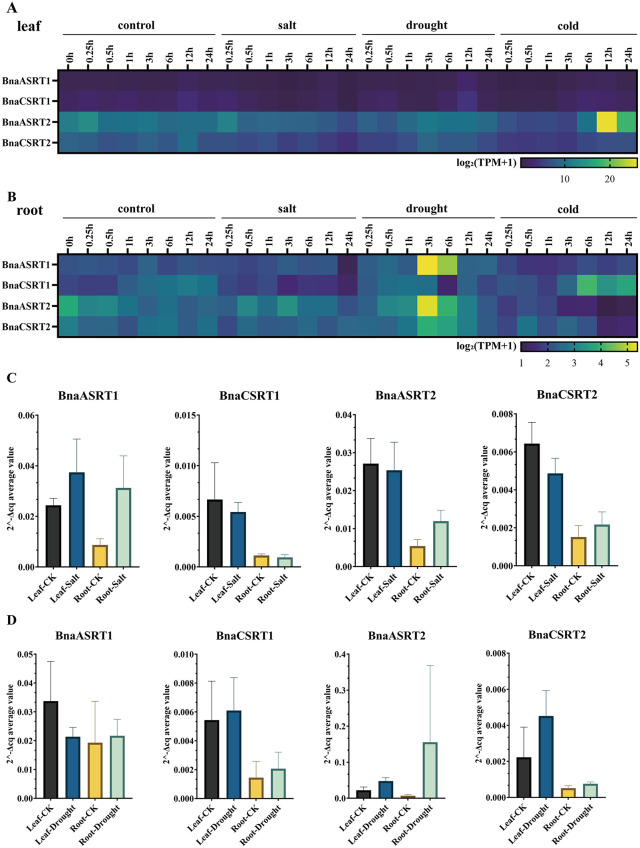
Expression profiles of the rapeseed *BnaSRTs* under different abiotic stresses, and qRT-PCR validation. These expression profiles pertain respectively to the leaves **(A)** and the roots **(B)** at different time points under different abiotic stresses (salt, drought, freezing, cold, head and osmotic). The gradient of colors from blue to yellow represents the degree of the expression value [log2(TPM + 1)] provided by the color scale. qRT-PCR validation was performed using materials from high salt treatment (1.3% NaCl, 2 days) **(C)**, drought treatment (20% PEG, 1 day) **(D)**, and untreated rapeseed. The horizontal axis represents the treatment method and location of the material, the vertical axis represents the gene expression levels (2^-ΔCq average value), and the subtitles indicate the genes being measured.

Based on the characterized functions of *SRTs* in other species and promoter element predictions, *BnaSRTs* are hypothesized to play critical roles in stress responses. In this study, transcriptome dynamics of *BnaSRTs* under diverse abiotic stresses (salt, drought, freezing, cold, heat, and osmotic stress) were analyzed using publicly available datasets from BnIR (https://yanglab.hzau.edu.cn/BnIR/) (Fig 7, S5 Table, and S9 Fig).

In leaf tissues, high-salt stress induced a time-dependent downregulatory trend in the expression of *BnaASRT2* and *BnaCSRT2*. Under drought stress, *BnaASRT2* and *BnaCSRT2* expression exhibited a biphasic pattern: initial upregulation followed by downregulation over time, with a critical turning point at 3 h. Cold stress triggered a similar biphasic trend (initial upregulation then downregulation) for both gene subgroups, with *BnaASRT1*/*BnaCSRT1* and *BnaASRT2*/*BnaCSRT2* reaching their turning points at 6 h and 12 h, respectively (Fig 7A, S5 Table). Osmotic stress consistently suppressed the expression of all BnaSRTs over time (S9 Fig, S5 Table).

In root tissues, high-salt stress led to a progressive downregulation of *BnaASRT1* and *BnaCSRT1*. Drought stress elicited distinct responses: *BnaASRT1*, *BnaASRT2*, and *BnaCSRT2* showed early upregulation (peaking at 3 h) followed by downregulation, whereas *BnaCSRT1* exhibited the opposite pattern with a turning point at 6 h. Under cold stress, *BnaASRT1*/*BnaCSRT1* expression displayed a recovery trend (initial downregulation then upregulation), in contrast to the sustained downregulation of *BnaASRT2*/*BnaCSRT2* (Fig 7B, S5 Table). Freezing stress induced a transient surge at 6 h in *BnaASRT1*, *BnaASRT2*, and *BnaCSRT2* (following initial downregulation), while *BnaCSRT1* remained downregulated throughout. Heat stress triggered a triphasic response in *BnaASRT1*/*BnaCSRT1*: downregulation, upregulation,(at 6 h) then re-downregulation. Osmotic stress led to consistent downregulation of *BnaASRT1* and *BnaCSRT1* over time (S9 Fig, S5 Table). The detection of the expression levels of *BnaSRTs* under different stress conditions also validated these results (Figs 7C and 7D). These results indicated that *BnaSRTs* responded to different stresses with distinct patterns, suggesting that BnaSRTs may play regulatory roles under various stress conditions.

### Expression profiles of *BnaSRTs* in response to different hormone treatment

**Fig 8:**
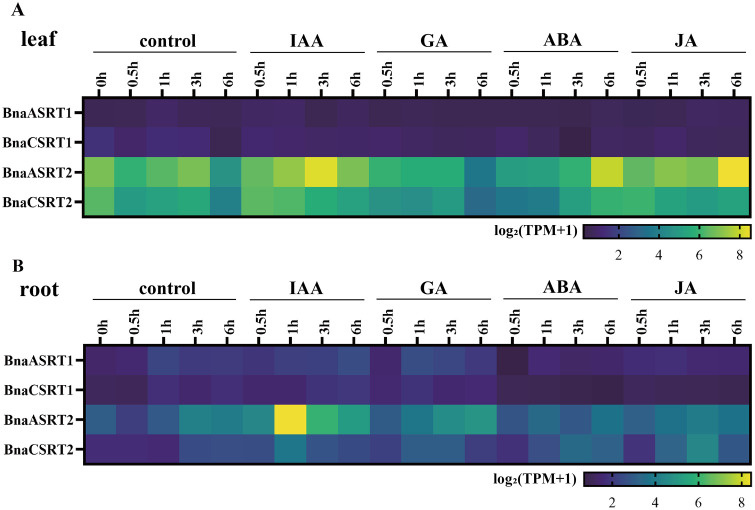
Expression profiles of the rapeseed *BnaSRTs* in response to different hormone treatment. These expression profiles pertain respectively to the leaves **(A)** and the roots **(B)** at different time points in response to different hormone treatment (IAA, ACC, GA, ABA, TZ, JA and BL). The gradient of colors from blue to yellow represents the degree of the expression value [log_2_(TPM + 1)] provided by the color scale.

Given the identification of multiple hormone-responsive elements in the *BnaSRT* promoters during our promoter element analysis, we further examined the expression dynamics of *BnaSRTs* in rapeseed following treatments with various hormones (IAA, ACC, GA, ABA, TZ, JA, and BL) using publicly available transcriptome datasets from BnIR (Fig 8, S6 Table and S10 Fig).

In leaf tissues, IAA treatment induced a biphasic expression pattern in *BnaASRT2* over time: initial upregulation followed by downregulation, with a critical turning point at 3 h. Under GA treatment, *BnaASRT2* and *BnaCSRT2* expression exhibited a sustained downregulatory trend over time. ABA treatment triggered a progressive upregulation of *BnaASRT2* and *BnaCSRT2* expression, while JA treatment led to a time-dependent upregulation specifically in *BnaASRT2* (Fig 8A). ACC treatment elicited a biphasic response in *BnaASRT2* expression (initial upregulation followed by downregulation) with a turning point at 1 h; similarly, TZ treatment induced a biphasic expression trend in *BnaASRT2* over time (S6 Table, S10 Fig).

In root tissues, IAA treatment induced a biphasic response in both *BnaASRT2* and *BnaCSRT2* expression, characterized by initial upregulation followed by downregulation with a turning point at 1 h (Fig 8B, S6 Table). Overall, *BnaSRT2* displayed more pronounced expression alterations in response to hormone treatments, which was consistent with the distribution of hormone-responsive elements in its promoter region.

## Discussion

As key epigenetic regulators, histone deacetylases (HDACs) modulate chromatin structure and gene expression via acetyl group removal from histone tails, thereby exerting pivotal functions in plant growth, development, and stress responses [50,51]. Within the HDAC superfamily, SIR2 family is defined by its conserved NAD ⁺ -dependent deacetylase activity. Having been extensively studied in yeast, animals, and model plants, This family is involved in a variety of biological processes, such as chromatin silencing, senescence, and stress tolerance. [52,53]. However, systematic identification and functional characterization of the SIR2 family in *Brassica napus* L. (rapeseed)-a globally important oilseed crop with high economic value-remain limited. In this study, we performed genome-wide identification of SIR2 family members in rapeseed, analyzed their phylogenetic relationships, structural features, and expression patterns, and provided insights into their potential roles in regulating the biological processes of rapeseed.

Genome-wide screening identified 4 SIR2 family members in rapeseed, which clustered into 2 distinct subgroups based on phylogenetic analysis (Fig 2B). This number exceeds that in *Arabidopsis thaliana* (2 members) [54] and *Oryza sativa* (rice, 2 members) [55], a discrepancy presumably linked to rapeseed’s allopolyploid genome (AACC). Rapeseed originated from the hybridization and subsequent polyploidization of *Brassica rapa* (AA genome) and *Brassica oleracea* (CC genome) [56]. Synteny analysis further confirmed that most *BnaSRT* members have orthologous counterparts in *B. rapa* and *B. oleracea* (Fig 2D), suggesting that polyploidization likely drove the expansion of the SIR2 family in rapeseed. Such expansion may have facilitated functional divergence, enabling rapeseed to adapt to more complex environmental niches during evolution.

All identified BnaSRTs proteins contain the conserved SIR2 domain (PF02146), which harbors the catalytic core essential for NAD ⁺ -dependent deacetylase activity [57] (Fig 3A). The high conservation of this domain across subgroups indicates that the core enzymatic functions of SIR2 proteins are evolutionarily conserved in rapeseed, consistent with observations in other plant species [54,55]. Despite strong conservation in their coding sequences, *BnaSRTs* genes exhibit substantial diversity in their upstream promoter regions: cis-acting elements differ markedly not only between *SRT1* and *SRT2* subgroups but also between homologous genes derived from different subgenomes (Fig 3D). This divergence points to functional specialization and regulatory recombination during evolution, supporting the notion that the expansion of the rapeseed SIR2 family is accompanied by both functional conservation and diversification.

Furthermore, molecular docking and inhibitor-based assays of potential modification sites revealed that rapeseed BnaSRTs target multiple deacetylation sites (Fig 4C and 5A). In contrast, most reported SRT-mediated modifications in other species are restricted to H3K9ac [11,18]. This suggests potential functional diversification of rapeseed BnaSRTs. Given the current focus on H3K9ac with limited research on other modification sites, substantial scope remains for exploring BnaSRTs' functions. Beyond histone deacetylation, emerging evidence indicates that SIR2 family proteins possess broader deacylation activities. In rice, OsSRT1 and OsSRT2 mediate the removal of lysine 2-hydroxyisobutyrylation (Khib) marks to regulate resistance against the pathogen Ustilaginoidea virens [58]. They also function in removing β-hydroxybutyrylation (Kbhb) modifications to modulate immune responses [11], while OsSRT2 is involved in crotonylation (Kcr) removal [59]. In wheat, TaSRT1 negatively regulates cold tolerance by decrotonylating TaPGK, thereby inhibiting its accumulation [60]. In cotton, GhSRT1 suppresses Verticillium wilt resistance by removing Khib and succinylation (Ksuc) marks [61]. BnSRTs may similarly possess these biochemical activities and exert specific biological functions in rapeseed.

Gene expression patterns are tightly linked to biological function [62]. Tissue-specific expression analysis revealed distinct expression profiles of *BnSRTs* across roots, stems, leaves, buds, flowers, siliques, and seeds (Fig 6). For example, high expression of *BnaASRT2* in siliques suggests potential involvement in seed development or nutrient accumulation-a critical agronomic trait in rapeseed. Its preferential expression in roots also implies roles in root system establishment, which is vital for nutrient uptake and stress tolerance [63]. These tissue-specific patterns share both similarities and differences with those in *Arabidopsis* [5], reflecting conserved and species-specific regulatory roles of the SIR2 family in plant development. Notably, *BnSRTs* members exhibit dynamic expression responses to abiotic stresses (e.g., drought, salt, cold) and hormone treatments (e.g., ABA, JA, GA) (S7 and S8 Figs). For instance, *BnaASRT1* and *BnaASRT2* are significantly upregulated in roots under drought stress; *BnaASRT2* and *BnaCSRT1* are induced by cold treatment in leaves and roots, respectively; and ABA-a key stress hormone-strongly activates *BnaASRT2*, pointing to potential involvement in ABA-mediated stress signaling. Furthermore, the analysis of the promoter elements of *BnaSRTs* also revealed the presence of multiple cis-acting elements that respond to abiotic stresses and hormones. These findings align with previous reports that plant SIR2 regulate stress responses through epigenetic modulation of stress-related gene expression [64,65]. For rapeseed, which frequently encounters environmental stresses during cultivation, these stress-responsive *BnaSRTs* represent promising candidates for improving stress tolerance via epigenetic engineering.

In yeast and animals, the SIR2 family is well-characterized for roles in lifespan regulation, DNA repair, and metabolic homeostasis [66]. In plants, accumulating evidence supports their involvement in transposon silencing, flowering time control, and environmental adaptation [54,67]. This study extends these insights to rapeseed, a polyploid crop with a complex genome. The expansion of the BnaSRT family and its subgroup-specific structural features suggest adaptation to regulating polyploidy-associated processes, such as genome dosage balance or subgenome expression dominance [68], expression under stress, indicating functional redundancy, while others show subgenome-specific expression, suggesting neofunctionalization. Compared with SIR2 families in diploid relatives (*B. rapa* and *B. oleracea*), the larger number of BnaSRT members and their diverse expression patterns highlight polyploidization' s role in shaping rapeseed' s epigenetic regulatory network. This expansion likely enhanced environmental adaptation flexibility-a trait critical for rapeseed' s domestication and widespread cultivation [69].

While this study provides a comprehensive framework for understanding the BnaSRTs family, limitations remain. First, functional inferences based on expression and structural features require experimental validation. Future studies employing CRISPR/Cas9-mediated knockout or overexpression of key BnaSRT members will clarify their roles in rapeseed development and stress responses. Second, the molecular mechanisms underlying BnaSRT-mediated regulation-including target genes, interacting partners (e.g., chromatin modifiers or transcription factors), and post-translational modifications-remain unclear. Chromatin immunoprecipitation sequencing (ChIP-seq) or yeast two-hybrid assays could identify downstream targets and protein interactors, elucidating their epigenetic regulatory networks. Third, responses of BnaSRTs to biotic stresses (e.g., pathogens) or agronomic cues (e.g., nutrient deficiency) were not investigated; given their potential role in integrating environmental signals, this warrants further research.

In summary, through genome-wide identification and expression profiling, this study reveals evolutionary expansion, structural conservation, and diverse developmental/stress-responsive expression patterns of the rapeseed SIR2 family. These findings advance our understanding of epigenetic regulation in polyploid crops and provide valuable candidate genes for improving rapeseed agronomic traits via epigenetic manipulation.

## Conclusions

This study is the first systematic identification and characterization of the *SRTs* gene family in *Brassica napus*, identifying four members (*BnaASRT1/2*, *BnaCSRT1/2*) and analyzing their structural, evolutionary, and functional traits. Evolutionarily, rapeseed SRTs are highly conserved with homologs in *Arabidopsis*, rice, and humans (retaining the NAD ⁺ -dependent deacetylase-related SIR2 domain) and were stably inherited during polyploidization. Structurally, they share conserved gene architectures and motifs, while divergent promoter cis-elements (e.g., stress/hormone-responsive) imply SRT1/SRT2 subgroup functional specialization. Functionally, BnaSRTs mediate histone deacetylation (binding H3K9/14/23ac and H4K5ac strongly) and regulate photosynthesis, metabolism, and stress pathways (supported by inhibitor and transcriptome analyses). They show tissue-specific expression (e.g., seed maturation) and respond to abiotic stresses/hormones. Overall, the study reveals SRTs' evolutionary conservation and functional diversity in rapeseed. BnaSRTs (especially BnaSRT2s) are promising for epigenetic engineering to improve stress tolerance and agronomic traits, with future studies needed to clarify mechanisms for breeding application.

## Supporting information

S1 Raw images Uncropped, original and complete Western Blot images. (PDF)

S1 Fig Protein sequence alignment with labeled SIR2 domain. (TIF)

S2 Fig KEGG pathway enrichment analysis results of BnaSRT proteins. (TIF)

S3 Fig 3D structure diagram of histone acetylation modifications. (TIF)

S4 Fig 2D molecular docking diagram of BnaASRT1 and 5 types of histone acetylation modifications. (TIF)

S5 Fig 2D molecular docking diagram of BnaCSRT1 and 5 types of histone acetylation modifications. (TIF)

S6 Fig 2D molecular docking diagram of BnaASRT2 and 5 types of histone acetylation modifications. (TIF)

S7 Fig 2D molecular docking diagram of BnaCSRT2 and 5 types of histone acetylation modifications. (TIF)

S8 Fig WB full results. The red box marks the part that is cropped and used.
(TIF)

S9 Fig Expression profiles of the rapeseed BnaSRTs under different abiotic stresses. These expression profiles pertain respectively to the leaves (A) and the roots (B) at different time points under different abiotic stresses (freezing, heat and osmotic).
(TIF)

S10 Fig Expression profiles of the rapeseed *BnaSRTs* in response to different hormone treatment. These expression profiles pertain respectively to the leaves (A) and the roots (B) at different time points in response to different hormone treatment (ACC, TZ and BL).
(TIF)

S1 Table The accession number for the genes used in this study. (XLSX)

S2 Table Detailed information about motifs. (XLSX)

S3 Table Detailed information about GO and KEGG pathway enrichment analysis results. (XLSX)

S4 Table Gene expression information in the tissues of rapeseed. (XLSX)

S5 Table Gene expression information in rapeseed during abiotic stress treatment. (XLSX)

S6 Table Gene expression information in rapeseed during hormone stress treatment. (XLSX)

S7 Table GO and KEGG enrichment analysis. (XLSX)

S8 Table Nucleotide sequences of primers used in this study. (XLSX)

## References

[1] Tahir MS, Tian L. HD2-type histone deacetylases: unique regulators of plant development and stress responses. Plant Cell Rep. 2021;40(9):1603–15. doi: 10.1007/s00299-021-02688-3 34041586

[2] Chang Y-N, Zhu C, Jiang J, Zhang H, Zhu J-K, Duan C-G. Epigenetic regulation in plant abiotic stress responses. J Integr Plant Biol. 2020;62(5):563–80. doi: 10.1111/jipb.12901 31872527

[3] Ma L, Xing L, Li Z, Jiang D. Epigenetic control of plant abiotic stress responses. J Genet Genomics. 2025;52(2):129–44. doi: 10.1016/j.jgg.2024.09.008 39322116

[4] Rothbart SB, Strahl BD. Interpreting the language of histone and DNA modifications. Biochim Biophys Acta. 2014;1839(8):627–43. doi: 10.1016/j.bbagrm.2014.03.001 24631868 PMC4099259

[5] Gray SG, Ekström TJ. The human histone deacetylase family. Exp Cell Res. 2001;262(2):75–83. doi: 10.1006/excr.2000.5080 11139331

[6] Kuo MH, Allis CD. Roles of histone acetyltransferases and deacetylases in gene regulation. Bioessays. 1998;20(8):615–26. doi: 10.1002/(SICI)1521-1878(199808)20:8<615::AID-BIES4>3.0.CO;2-H 9780836

[7] Chen X, Ding AB, Zhong X. Functions and mechanisms of plant histone deacetylases. Sci China Life Sci. 2020;63(2):206–16. doi: 10.1007/s11427-019-1587-x 31879846

[8] Li H, Chen Y, Dai Y, Yang L, Zhang S. Genome-wide identification and expression analysis of histone deacetylase and histone acetyltransferase genes in response to drought in poplars. BMC Genomics. 2024;25(1):657. doi: 10.1186/s12864-024-10570-1 38956453 PMC11218084

[9] Wang P, Su L, Cao L, Hu H, Wan H, Wu C, et al. AtSRT1 regulates flowering by regulating flowering integrators and energy signals in Arabidopsis. Plant Physiol Biochem. 2024;213:108841. doi: 10.1016/j.plaphy.2024.108841 38879987

[10] Obrecht A, Paneque M. Unraveling the Role of AtSRT2 in Energy Metabolism, Stress Responses, and Gene Expression during Osmotic Stress in Arabidopsis thaliana. Plants (Basel). 2024;13(5):711. doi: 10.3390/plants13050711 38475557 PMC10934544

[11] Zhang H, Zhao Y, Zhou D-X. Rice NAD+-dependent histone deacetylase OsSRT1 represses glycolysis and regulates the moonlighting function of GAPDH as a transcriptional activator of glycolytic genes. Nucleic Acids Res. 2017;45(21):12241–55. doi: 10.1093/nar/gkx825 28981755 PMC5716216

[12] Fang C, Zhang H, Wan J, Wu Y, Li K, Jin C, et al. Control of Leaf Senescence by an MeOH-Jasmonates Cascade that Is Epigenetically Regulated by OsSRT1 in Rice. Mol Plant. 2016;9:1366–78. 10.1016/j.molp.2016.07.007 27477683

[13] König A-C, Hartl M, Pham PA, Laxa M, Boersema PJ, Orwat A, et al. The Arabidopsis class II sirtuin is a lysine deacetylase and interacts with mitochondrial energy metabolism. Plant Physiol. 2014;164(3):1401–14. doi: 10.1104/pp.113.232496 24424322 PMC3938629

[14] Bruscalupi G, Di Micco P, Failla CM, Pascarella G, Morea V, Saliola M, et al. Arabidopsis thaliana sirtuins control proliferation and glutamate dehydrogenase activity. Plant Physiol Biochem. 2023;194:236–45. doi: 10.1016/j.plaphy.2022.11.007 36436414

[15] Zhang F, Wang L, Ko EE, Shao K, Qiao H. Histone deacetylases SRT1 and SRT2 Interact with ENAP1 to mediate ethylene-induced transcriptional repression. Plant Cell. 2018;30(1):153–66. doi: 10.1105/tpc.17.00671 29298835 PMC5810571

[16] Vall-Llaura N, Torres R, Lindo-García V, Muñoz P, Munné-Bosch S, Larrigaudière C, et al. PbSRT1 and PbSRT2 regulate pear growth and ripening yet displaying a species-specific regulation in comparison to other Rosaceae spp. Plant Sci. 2021;308:110925. doi: 10.1016/j.plantsci.2021.110925 34034873

[17] Yang M, Song C, He X, Wang L, He C, Yu H, et al. The new function of FaSRT2-1 protein in energy metabolism: Promoting strawberry fruit quality and ripening. Int J Biol Macromol. 2024;281(Pt 2):136199. doi: 10.1016/j.ijbiomac.2024.136199 39366613

[18] Liu X, Wei W, Zhu W, Su L, Xiong Z, Zhou M, et al. Histone deacetylase AtSRT1 links metabolic flux and stress response in *Arabidopsis*. Mol Plant. 2017;10(12):1510–22. doi: 10.1016/j.molp.2017.10.010 29107034

[19] Wang C, Gao F, Wu J, Dai J, Wei C, Li Y. Arabidopsis putative deacetylase AtSRT2 regulates basal defense by suppressing PAD4, EDS5 and SID2 expression. Plant Cell Physiol. 2010;51(8):1291–9. doi: 10.1093/pcp/pcq087 20573705 PMC2920754

[20] Tang W-S, Zhong L, Ding Q-Q, Dou Y-N, Li W-W, Xu Z-S, et al. Histone deacetylase AtSRT2 regulates salt tolerance during seed germination via repression of vesicle-associated membrane protein 714 (VAMP714) in Arabidopsis. New Phytol. 2022;234(4):1278–93. doi: 10.1111/nph.18060 35224735

[21] Huang L, Sun Q, Qin F, Li C, Zhao Y, Zhou D-X. Down-regulation of a SILENT INFORMATION REGULATOR2-related histone deacetylase gene, OsSRT1, induces DNA fragmentation and cell death in rice. Plant Physiol. 2007;144(3):1508–19. doi: 10.1104/pp.107.099473 17468215 PMC1914135

[22] Wang L, Lin Y, Hou G, Yang M, Peng Y, Jiang Y, et al. A histone deacetylase, FaSRT1-2, plays multiple roles in regulating fruit ripening, plant growth and stresses resistance of cultivated strawberry. Plant Cell Environ. 2024;47(6):2258–73. doi: 10.1111/pce.14885 38482979

[23] Wang Y, Zeng Q, Tian Y, Deng Q, Xiao R, Luo X, et al. The histone deacetylase SRT2 enhances the tolerance of chrysanthemum to low temperatures through the ROS scavenging system. Plant Physiol Biochem. 2024;207:108405. doi: 10.1016/j.plaphy.2024.108405 38354529

[24] Chang S, Yang Q, Chu W, Liu X, Li J, Liu Z, et al. Lysine deacetylase TaSRT1 mediates wheat drought tolerance by deacetylating TaDT-A to reduce its protein stability and transcriptional activity. Plant Biotechnol J. 2025;23(5):1650–67. doi: 10.1111/pbi.14613 39977256 PMC12018820

[25] Zhang N, Liu L, Li S, Wei Y, Ren W, Li M, et al. TaHSP18.6 and TaSRT1 interact to confer resistance to Fusarium crown rot by regulating the auxin content in common wheat. Proc Natl Acad Sci U S A. 2025;122(28):e2500029122. doi: 10.1073/pnas.2500029122 40632559 PMC12280888

[26] Goodstein DM, Shu S, Howson R, Neupane R, Hayes RD, Fazo J, et al. Phytozome: a comparative platform for green plant genomics. Nucleic Acids Res. 2012;40(Database issue):D1178-86. doi: 10.1093/nar/gkr944 22110026 PMC3245001

[27] Goldfarb T, Kodali VK, Pujar S, Brover V, Robbertse B, Farrell CM, et al. NCBI RefSeq: reference sequence standards through 25 years of curation and annotation. Nucleic Acids Res. 2024;53:D243–57. doi: 10.1093/nar/gkae1038 PMC11701664 39526381

[28] Yang Z, Wang S, Wei L, Huang Y, Liu D, Jia Y, et al. BnIR: a multi-omics database with various tools for *Brassica napus* research and breeding. Mol Plant. 2023;16(4):775–89. doi: 10.1016/j.molp.2023.03.007 36919242

[29] Wang J, Chitsaz F, Derbyshire MK, Gonzales NR, Gwadz M, Lu S, et al. The conserved domain database in 2023. Nucleic Acids Res. 2023;51(D1):D384–8. doi: 10.1093/nar/gkac1096 36477806 PMC9825596

[30] Wilkins MR, Gasteiger E, Bairoch A, Sanchez JC, Williams KL, Appel RD, et al. Protein identification and analysis tools in the ExPASy server. Methods Mol Biol. 1999;112:531–52. doi: 10.1385/1-59259-584-7:531 10027275

[31] Waterhouse A, Bertoni M, Bienert S, Studer G, Tauriello G, Gumienny R, et al. SWISS-MODEL: homology modelling of protein structures and complexes. Nucleic Acids Res. 2018;46(W1):W296–303. doi: 10.1093/nar/gky427 29788355 PMC6030848

[32] Delano WL. The PyMOL molecular graphics system. Protein Struct Funct Bioinform. 2002;33:442–54. http://dx.doi.org/.

[33] Sievers F, Wilm A, Dineen D, Gibson TJ, Karplus K, Li W, et al. Fast, scalable generation of high-quality protein multiple sequence alignments using Clustal Omega. Mol Syst Biol. 2011;7:539. doi: 10.1038/msb.2011.75 21988835 PMC3261699

[34] Tamura K, Stecher G, Kumar S. MEGA11: molecular evolutionary genetics analysis version 11. Mol Biol Evol. 2021;38(7):3022–7. doi: 10.1093/molbev/msab120 33892491 PMC8233496

[35] Letunic I, Bork P. Interactive Tree Of Life (iTOL) v5: an online tool for phylogenetic tree display and annotation. Nucleic Acids Res. 2021;49(W1):W293–6. doi: 10.1093/nar/gkab301 33885785 PMC8265157

[36] Lamesch P, Berardini TZ, Li D, Swarbreck D, Wilks C, Sasidharan R, et al. The Arabidopsis Information Resource (TAIR): improved gene annotation and new tools. Nucleic Acids Res. 2012;40(Database issue):D1202-10. doi: 10.1093/nar/gkr1090 22140109 PMC3245047

[37] Chen C, Wu Y, Li J, Wang X, Zeng Z, Xu J, et al. TBtools-II: a “one for all, all for one” bioinformatics platform for biological big-data mining. Mol Plant. 2023;16(11):1733–42. doi: 10.1016/j.molp.2023.09.010 37740491

[38] Sayers EW, Cavanaugh M, Clark K, Pruitt KD, Schoch CL, Sherry ST, et al. GenBank. Nucleic Acids Res. 2022;50(D1):D161–4. doi: 10.1093/nar/gkab1135 34850943 PMC8690257

[39] Hamilton JP, Li C, Buell CR. The rice genome annotation project: an updated database for mining the rice genome. Nucleic Acids Res. 2025;53(D1):D1614–22. doi: 10.1093/nar/gkae1061 39558187 PMC11701632

[40] Bailey TL, Johnson J, Grant CE, Noble WS. The MEME suite. Nucleic Acids Res. 2015;43(W1):W39-49. doi: 10.1093/nar/gkv416 25953851 PMC4489269

[41] Lescot M, Déhais P, Thijs G, Marchal K, Moreau Y, Van de Peer Y, et al. PlantCARE, a database of plant cis-acting regulatory elements and a portal to tools for in silico analysis of promoter sequences. Nucleic Acids Res. 2002;30(1):325–7. doi: 10.1093/nar/30.1.325 11752327 PMC99092

[42] Szklarczyk D, Kirsch R, Koutrouli M, Nastou K, Mehryary F, Hachilif R, et al. The STRING database in 2023: protein-protein association networks and functional enrichment analyses for any sequenced genome of interest. Nucleic Acids Res. 2023;51(D1):D638–46. doi: 10.1093/nar/gkac1000 36370105 PMC9825434

[43] Burley SK, Bhatt R, Bhikadiya C, Bi C, Biester A, Biswas P, et al. Updated resources for exploring experimentally-determined PDB structures and computed structure models at the RCSB protein data bank. Nucleic Acids Res. 2025;53(D1):D564–74. doi: 10.1093/nar/gkae1091 39607707 PMC11701563

[44] Morris GM, Huey R, Lindstrom W, Sanner MF, Belew RK, Goodsell DS, et al. AutoDock4 and AutoDockTools4: automated docking with selective receptor flexibility. J Comput Chem. 2009;30(16):2785–91. doi: 10.1002/jcc.21256 19399780 PMC2760638

[45] Trott O, Olson AJ. AutoDock Vina: improving the speed and accuracy of docking with a new scoring function, efficient optimization, and multithreading. J Comput Chem. 2010;31(2):455–61. doi: 10.1002/jcc.21334 19499576 PMC3041641

[46] Diedrich K, Krause B, Berg O, Rarey M. PoseEdit: enhanced ligand binding mode communication by interactive 2D diagrams. J Comput Aided Mol Des. 2023;37(10):491–503. doi: 10.1007/s10822-023-00522-4 37515714 PMC10440272

[47] Du Z, Zhou X, Ling Y, Zhang Z, Su Z. agriGO: a GO analysis toolkit for the agricultural community. Nucleic Acids Res. 2010;38(Web Server issue):W64-70. doi: 10.1093/nar/gkq310 20435677 PMC2896167

[48] Mao X, Cai T, Olyarchuk JG, Wei L. Automated genome annotation and pathway identification using the KEGG Orthology (KO) as a controlled vocabulary. Bioinformatics. 2005;21(19):3787–93. doi: 10.1093/bioinformatics/bti430 15817693

[49] Hu J, He B, Bhargava S, Lin H. A fluorogenic assay for screening Sirt6 modulators. Org Biomol Chem. 2013;11(32):5213–6. doi: 10.1039/c3ob41138a 23839075 PMC3756594

[50] Ageeva-Kieferle A, Rudolf EE, Lindermayr C. Redox-dependent chromatin remodeling: a new function of nitric oxide as architect of chromatin structure in plants. Front Plant Sci. 2019;10:625. doi: 10.3389/fpls.2019.00625 31191565 PMC6546728

[51] Chinnusamy V, Zhu J-K. Epigenetic regulation of stress responses in plants. Curr Opin Plant Biol. 2009;12(2):133–9. doi: 10.1016/j.pbi.2008.12.006 19179104 PMC3139470

[52] Imai S, Armstrong CM, Kaeberlein M, Guarente L. Transcriptional silencing and longevity protein Sir2 is an NAD-dependent histone deacetylase. Nature. 2000;403(6771):795–800. doi: 10.1038/35001622 10693811

[53] Hsu CC, et al. Sirtuins in plants: emerging roles in metabolism, stress responses, and development. J Experim Bot. 2018;69:3629–40.

[54] Shahbazian MD, Grunstein M. Functions of sirtuins in yeast. Ann Rev Biochem. 2007;76:275–99. 10.1146/annurev.biochem.76.052705.162114 17362198

[55] Huang Y. Genome-wide analysis of the SIR2 family in rice and their expression under abiotic stress. BMC Genom. 2020;21:1–15.

[56] Chalhoub B, Denoeud F, Liu S, Parkin IAP, Tang H, Wang X, et al. Plant genetics. Early allopolyploid evolution in the post-Neolithic *Brassica napus* oilseed genome. Science. 2014;345(6199):950–3. doi: 10.1126/science.1253435 25146293

[57] Frye RA. Phylogenetic classification of prokaryotic and eukaryotic Sir2-like proteins. Biochem Biophys Res Commun. 2000;273(2):793–8. doi: 10.1006/bbrc.2000.3000 10873683

[58] Chen X, Xu Q, Duan Y, Liu H, Chen X, Huang J, et al. Ustilaginoidea virens modulates lysine 2-hydroxyisobutyrylation in rice flowers during infection. J Integr Plant Biol. 2021;63(10):1801–14. doi: 10.1111/jipb.13149 34245484

[59] Lu Y, Xu Q, Liu Y, Yu Y, Cheng Z-Y, Zhao Y, et al. Dynamics and functional interplay of histone lysine butyrylation, crotonylation, and acetylation in rice under starvation and submergence. Genome Biol. 2018;19(1):144. doi: 10.1186/s13059-018-1533-y 30253806 PMC6154804

[60] Zhang N, Wang S, Zhao S, Chen D, Tian H, Li J, et al. Global crotonylatome and GWAS revealed a TaSRT1-TaPGK model regulating wheat cold tolerance through mediating pyruvate. Sci Adv. 2023;9(19):eadg1012. doi: 10.1126/sciadv.adg1012 37163591 PMC10171821

[61] Chen B, Wang Z, Jiao M, Zhang J, Liu J, Zhang D, et al. Lysine 2-hydroxyisobutyrylation- and succinylation-based pathways act inside chloroplasts to modulate plant photosynthesis and immunity. Adv Sci (Weinh). 2023;10(27):e2301803. doi: 10.1002/advs.202301803 37492013 PMC10520639

[62] Wang X. Tissue-specific transcriptome analysis reveals key genes associated with oil accumulation in Brassica napus. Plant Biotech J. 2019;17:148–63.

[63] Smith LG, De Smet I. Root system architecture: from development to function. Curr Opin Plant Biol. 2012;15:111–7.22483403

[64] Kim J, et al. SIRT1-mediated deacetylation of histone H3K9 regulates drought stress response in Arabidopsis. Plant Cell. 2015;27.

[65] Zhang Y. Sir2-like histone deacetylases in wheat: genome-wide identification and expression analysis under abiotic stress. J Plant Physiol. 2021;261:153464.

[66] Guarente L. Sirtuins and the regulation of mitochondrial biology. Cold Spring Harbor Perspectives in Biology. 2013;5:a012986.

[67] Baur JA, Sinclair DA. Sirtuins in aging and metabolism. Nature. 2006;444:397–402.

[68] Edger PP. Subgenome dominance in an allopolyploid plant: biased gene expression and expression level dominance. Gen Biol. 2017;18:1–16.

[69] Chen S. Polyploidy in crop improvement: current status and future prospects. Plant Biotechnol J. 2020;18:1891–903.

