# Supplementary material for: Genome-wide identification and multi-dimensional functional characterization of the SIR2 family in Brassica napus L
Source: PLoS One. 2026 Jan 22;21(1):e0340688. doi: 10.1371/journal.pone.0340688 (PMC12826482; doi:10.1371/journal.pone.0340688)
Supplement: S1 Raw images — (PDF) [file pone.0340688.s001.pdf]

## H3

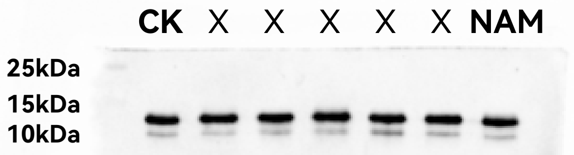


loading order: from left to right
imaging method: chemiluminescence
associated figure: fig5B

## H3

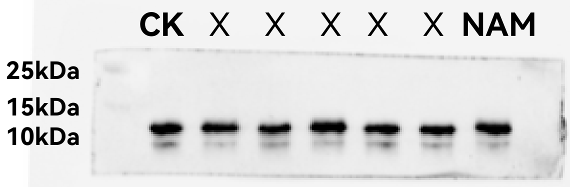


loading order: from left to right
imaging method: chemiluminescence
associated figure: fig5B

## H3K9ac

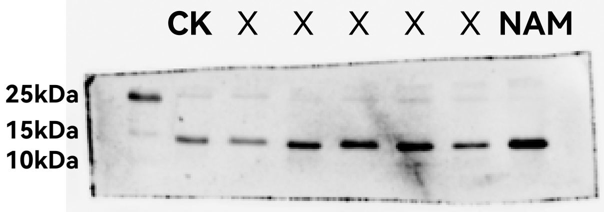


loading order: from left to right
imaging method: chemiluminescence
associated figure: fig5B

## H3K9ac

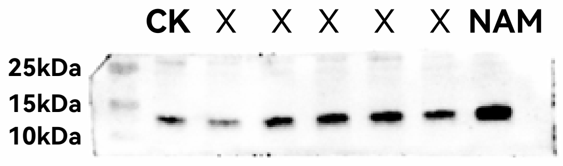


loading order: from left to right
imaging method: chemiluminescence
associated figure: fig5B

## H3K14ac

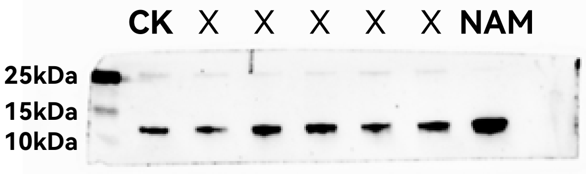


loading order: from left to right
imaging method: chemiluminescence
associated figure: fig5B

## H3K14ac

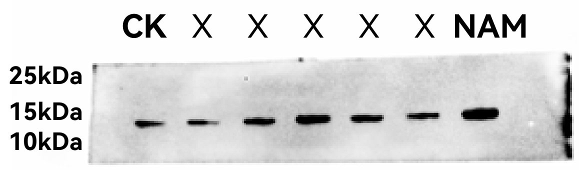


loading order: from left to right
imaging method: chemiluminescence
associated figure: fig5B

## H3K23ac

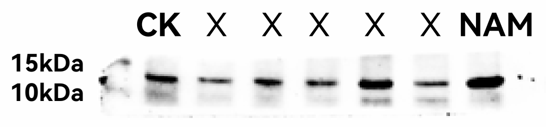


loading order: from left to right
imaging method: chemiluminescence
associated figure: fig5B

## H3K23ac

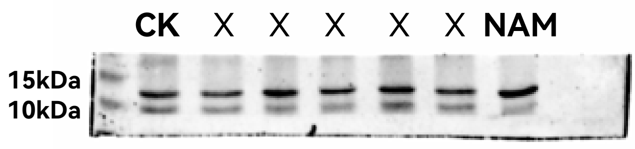


loading order: from left to right
imaging method: chemiluminescence
associated figure: fig5B

## H3K27ac

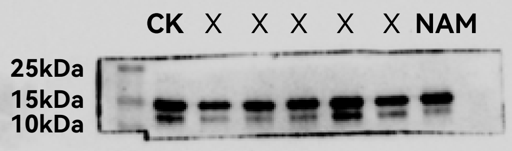


loading order: from left to right
imaging method: chemiluminescence
associated figure: fig5B

## H3K27ac

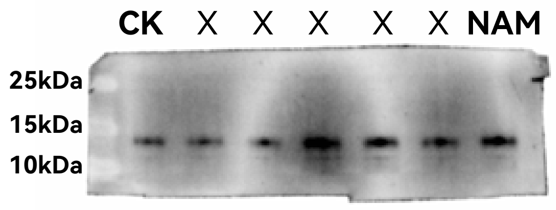


loading order: from left to right
imaging method: chemiluminescence
associated figure: fig5B

## H4K5ac

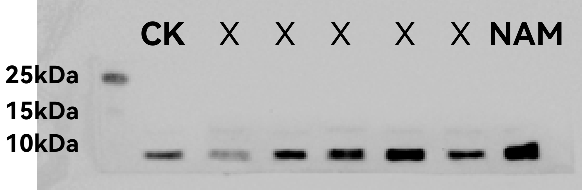


loading order: from left to right
imaging method: chemiluminescence
associated figure: fig5B

## H4K5ac

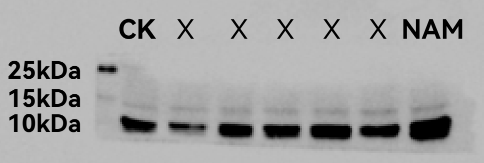


loading order: from left to right
imaging method: chemiluminescence
associated figure: fig5B
