# Supplementary figures and images for: Genome-wide identification and multi-dimensional functional characterization of the SIR2 family in Brassica napus L
Source: PLoS One. 2026 Jan 22;21(1):e0340688. doi: 10.1371/journal.pone.0340688 (PMC12826482; doi:10.1371/journal.pone.0340688)

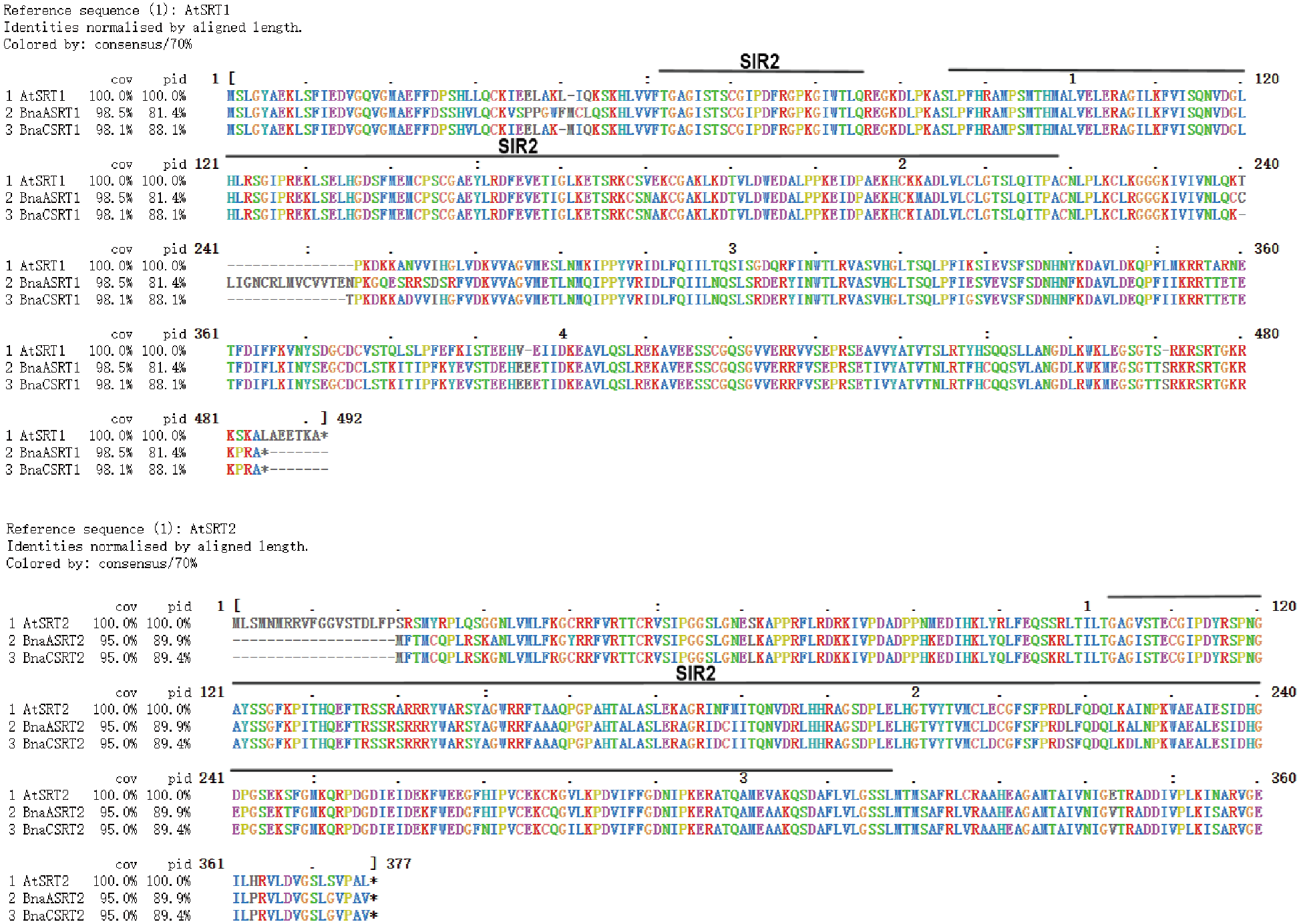

Supplement: S1 Fig — (TIF) [file pone.0340688.s002.tif]

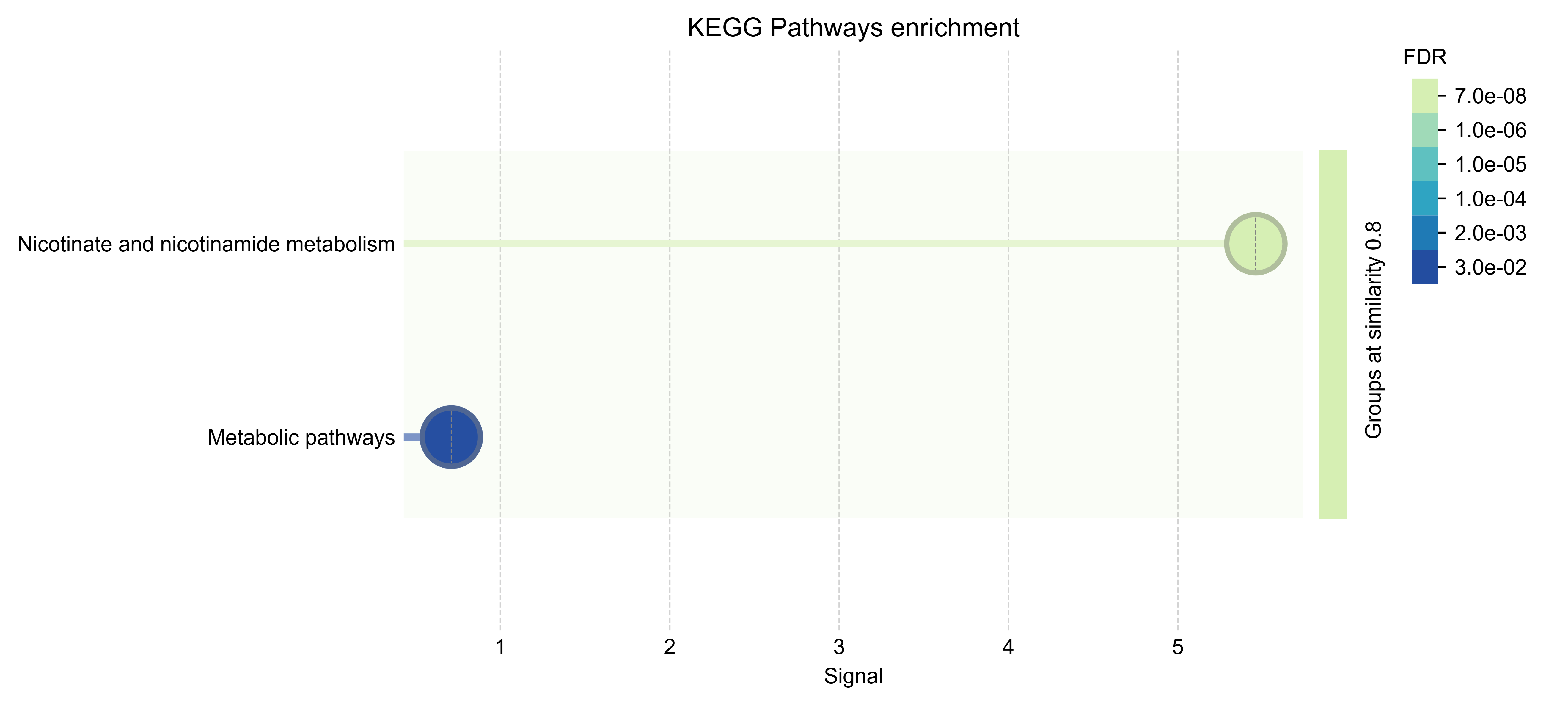

Supplement: S2 Fig — (TIF) [file pone.0340688.s003.tif]

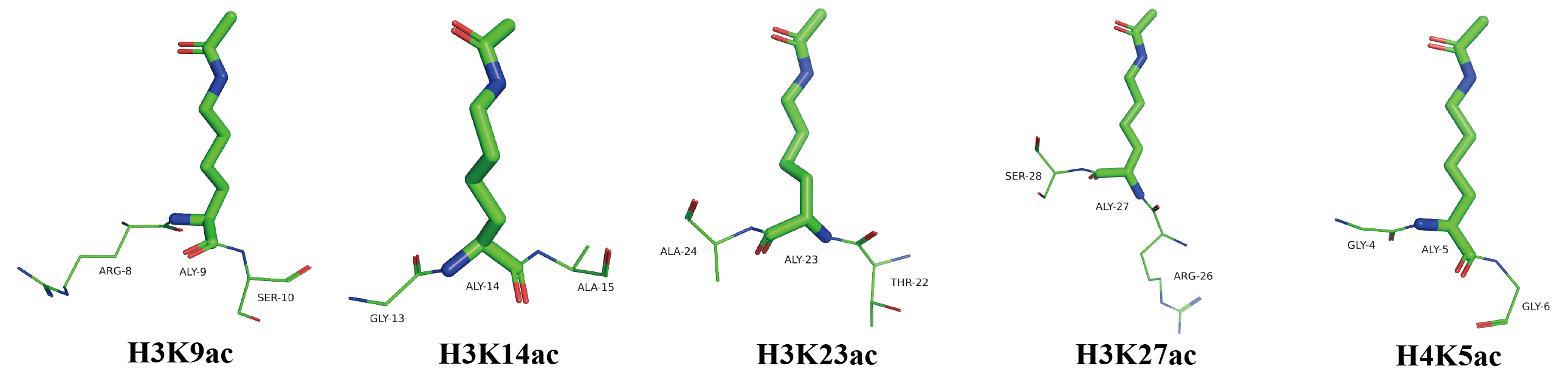

Supplement: S3 Fig — (TIF) [file pone.0340688.s004.tif]

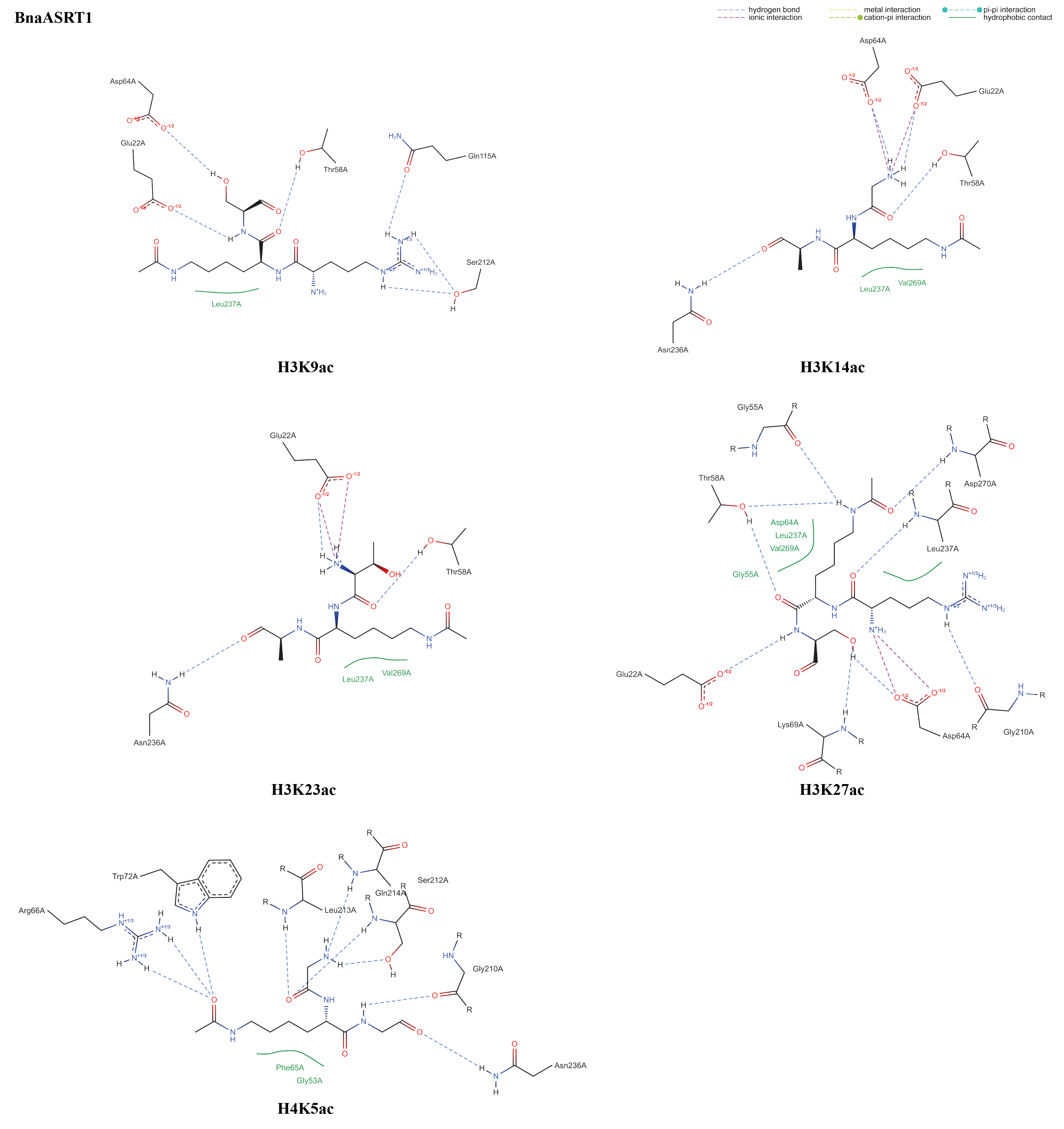

Supplement: S4 Fig — (TIF) [file pone.0340688.s005.tif]

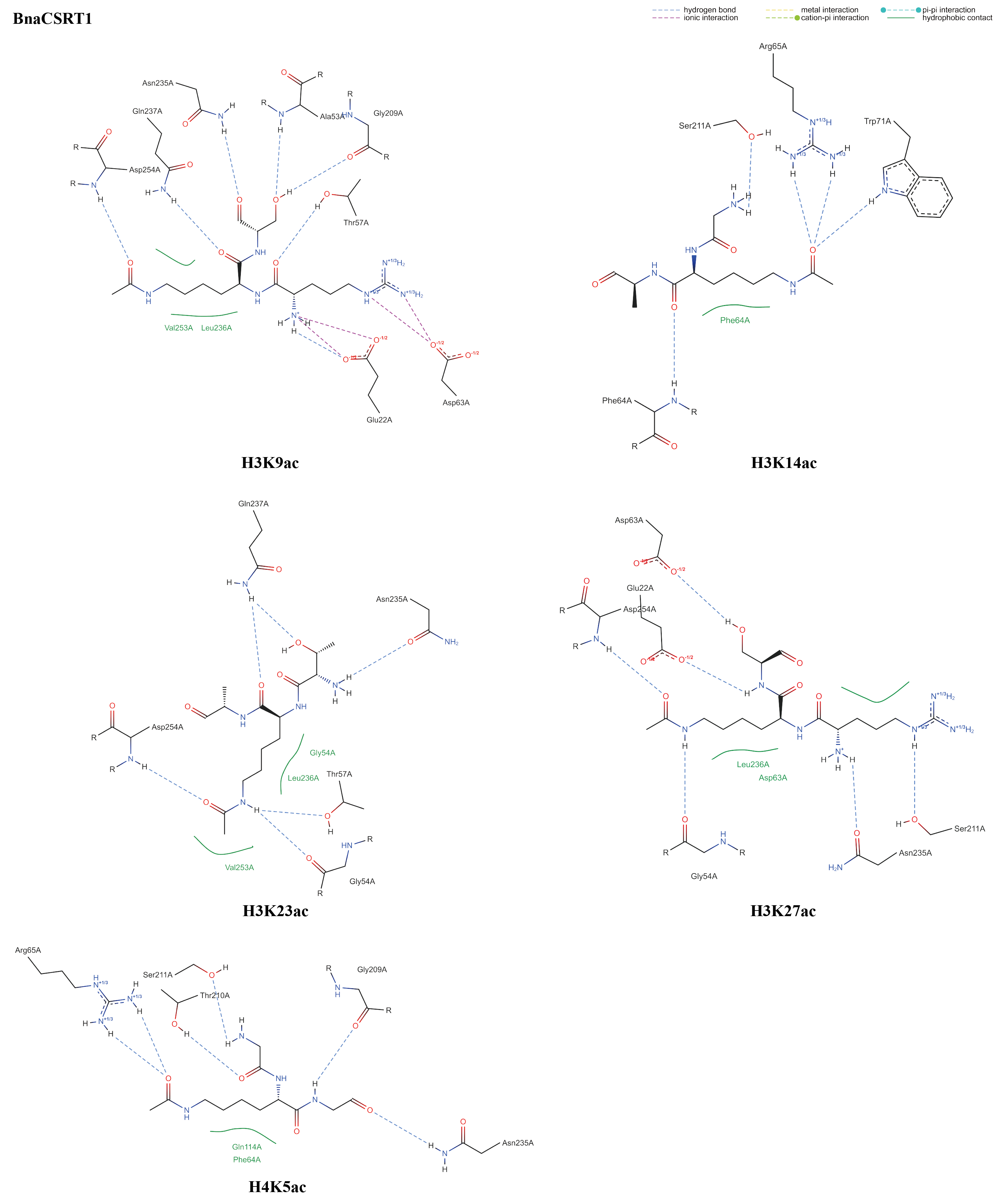

Supplement: S5 Fig — (TIF) [file pone.0340688.s006.tif]

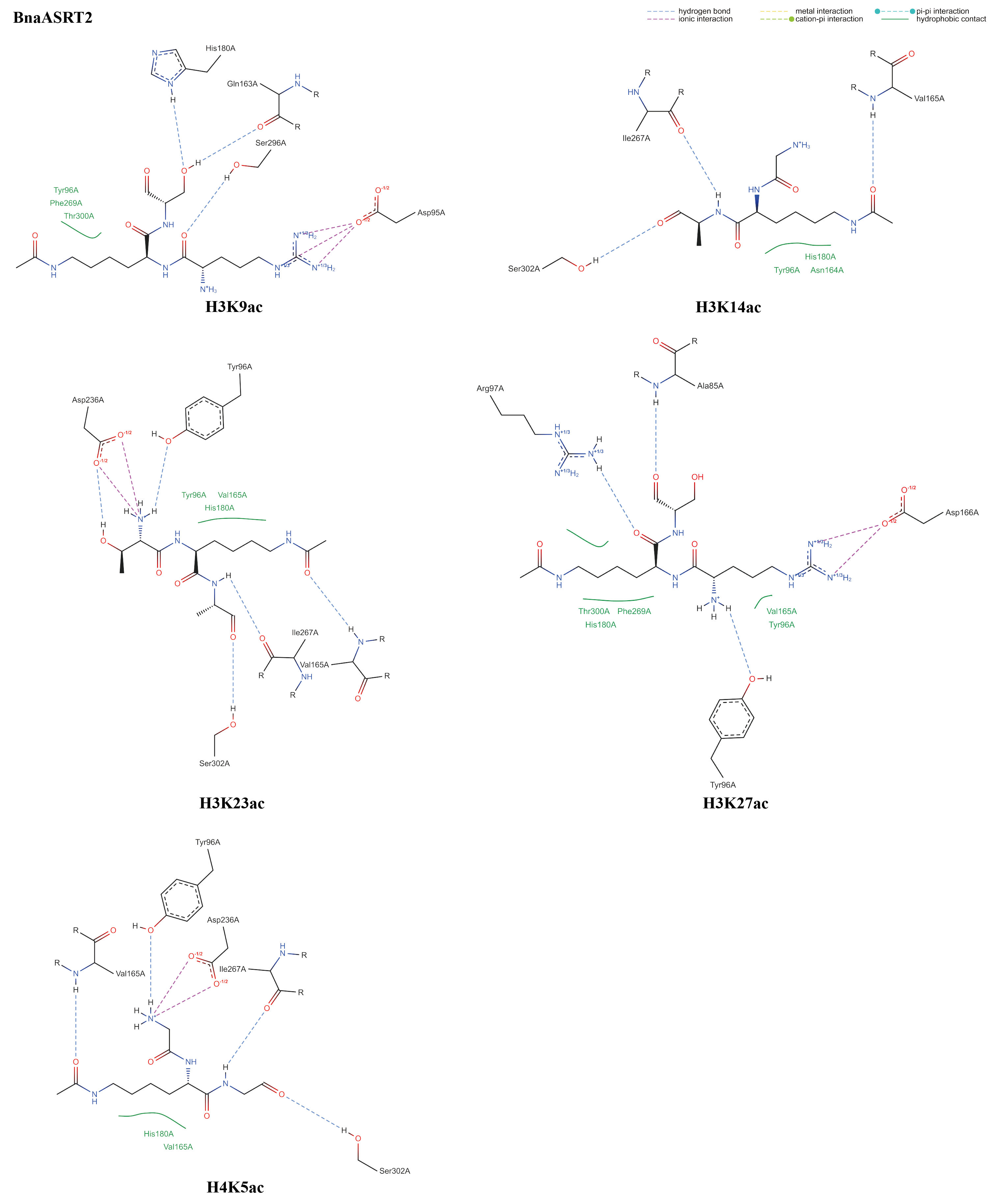

Supplement: S6 Fig — (TIF) [file pone.0340688.s007.tif]

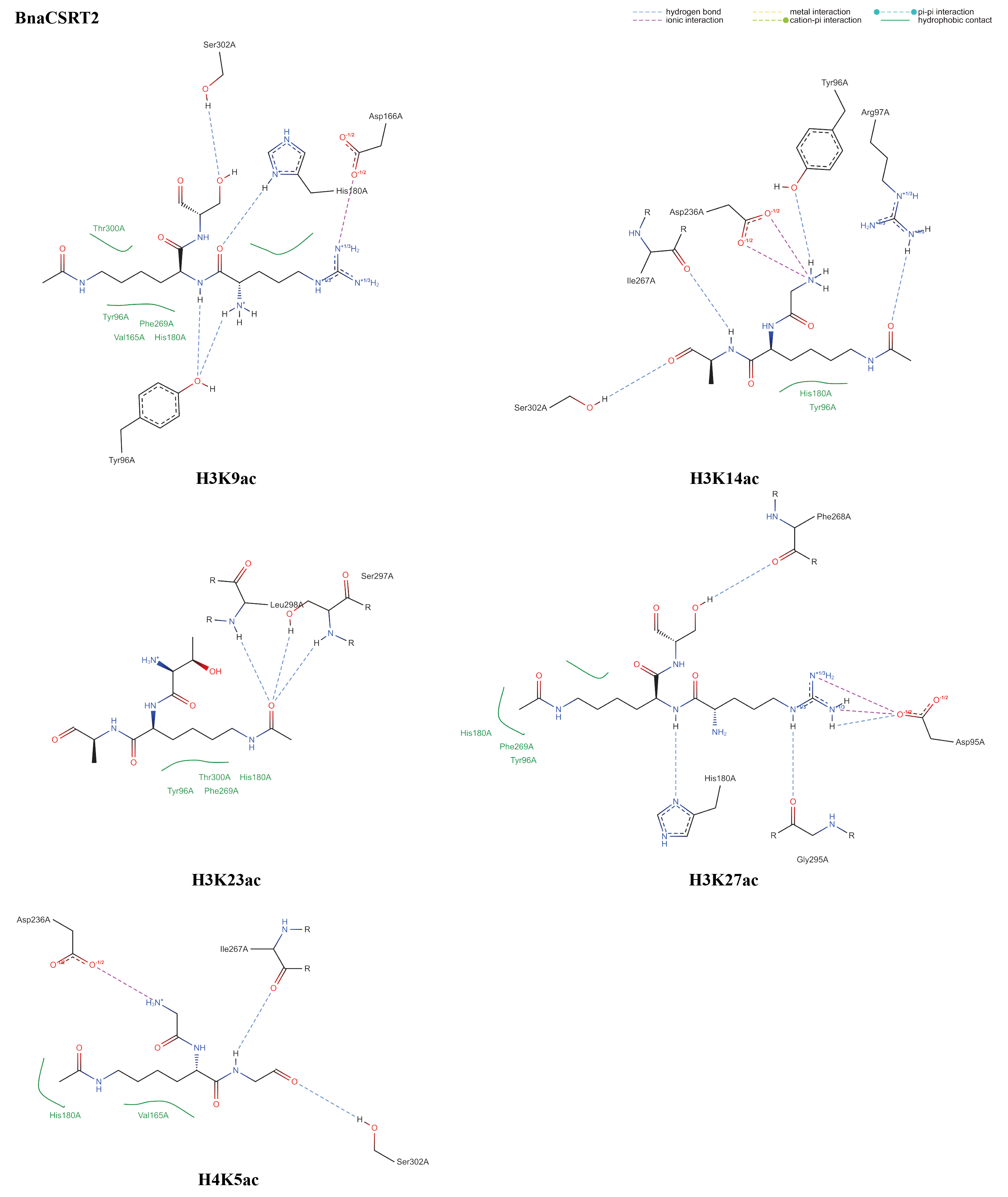

Supplement: S7 Fig — (TIF) [file pone.0340688.s008.tif]

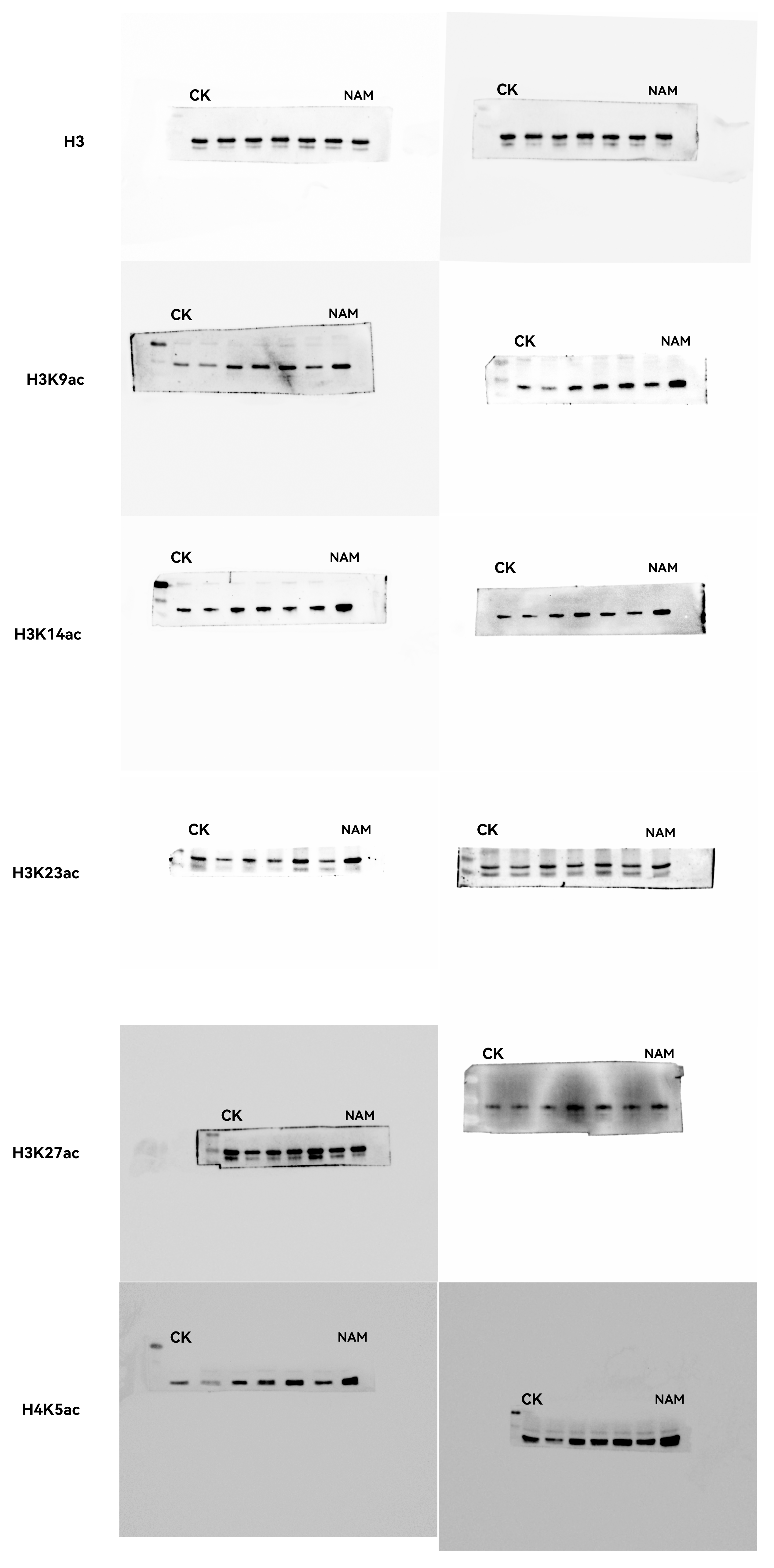

Supplement: S8 Fig — The red box marks the part that is cropped and used. (TIF) [file pone.0340688.s009.tif]

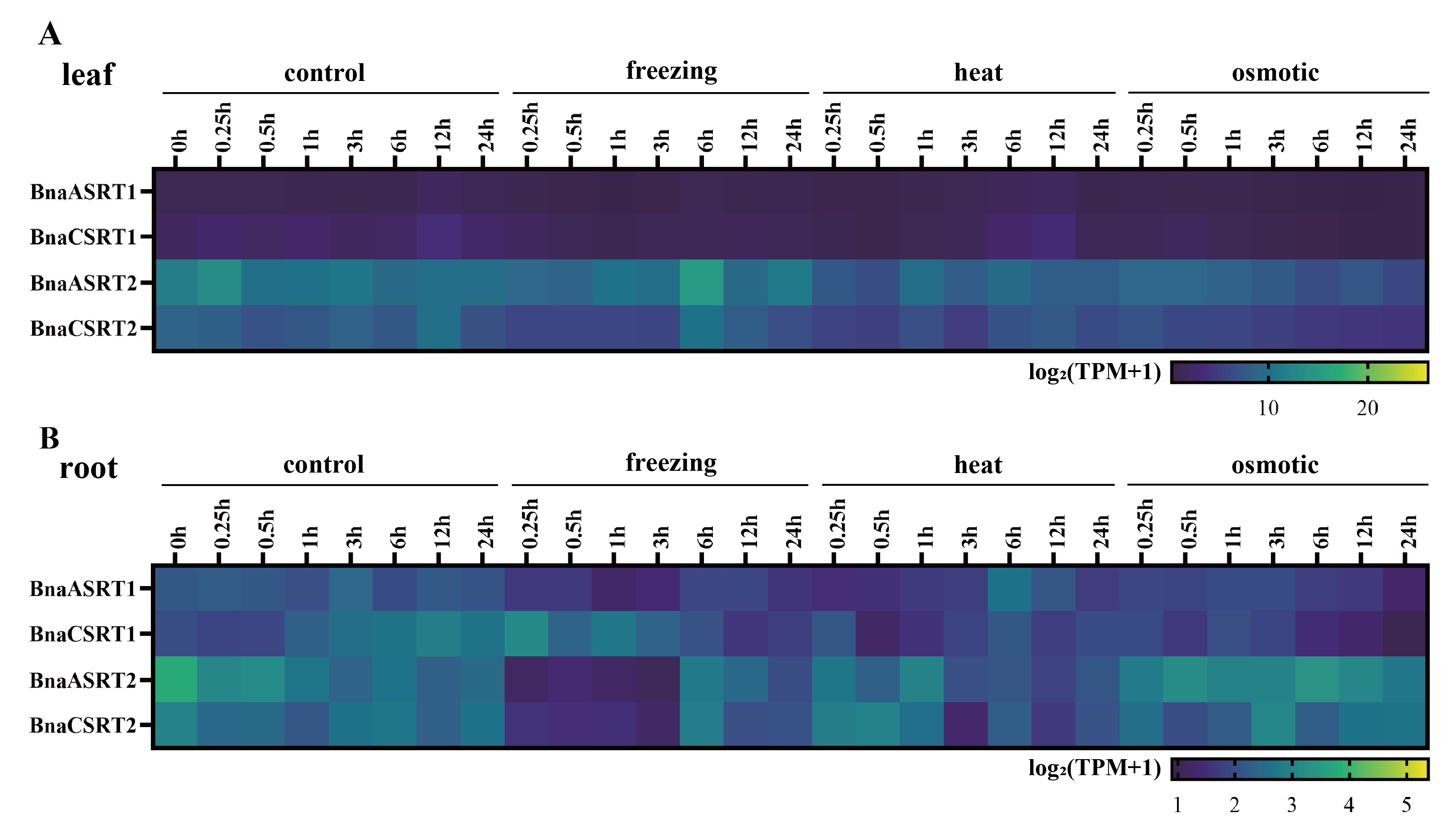

Supplement: S9 Fig — These expression profiles pertain respectively to the leaves (A) and the roots (B) at different time points under different abiotic stresses (freezing, heat and osmotic). (TIF) [file pone.0340688.s010.tif]

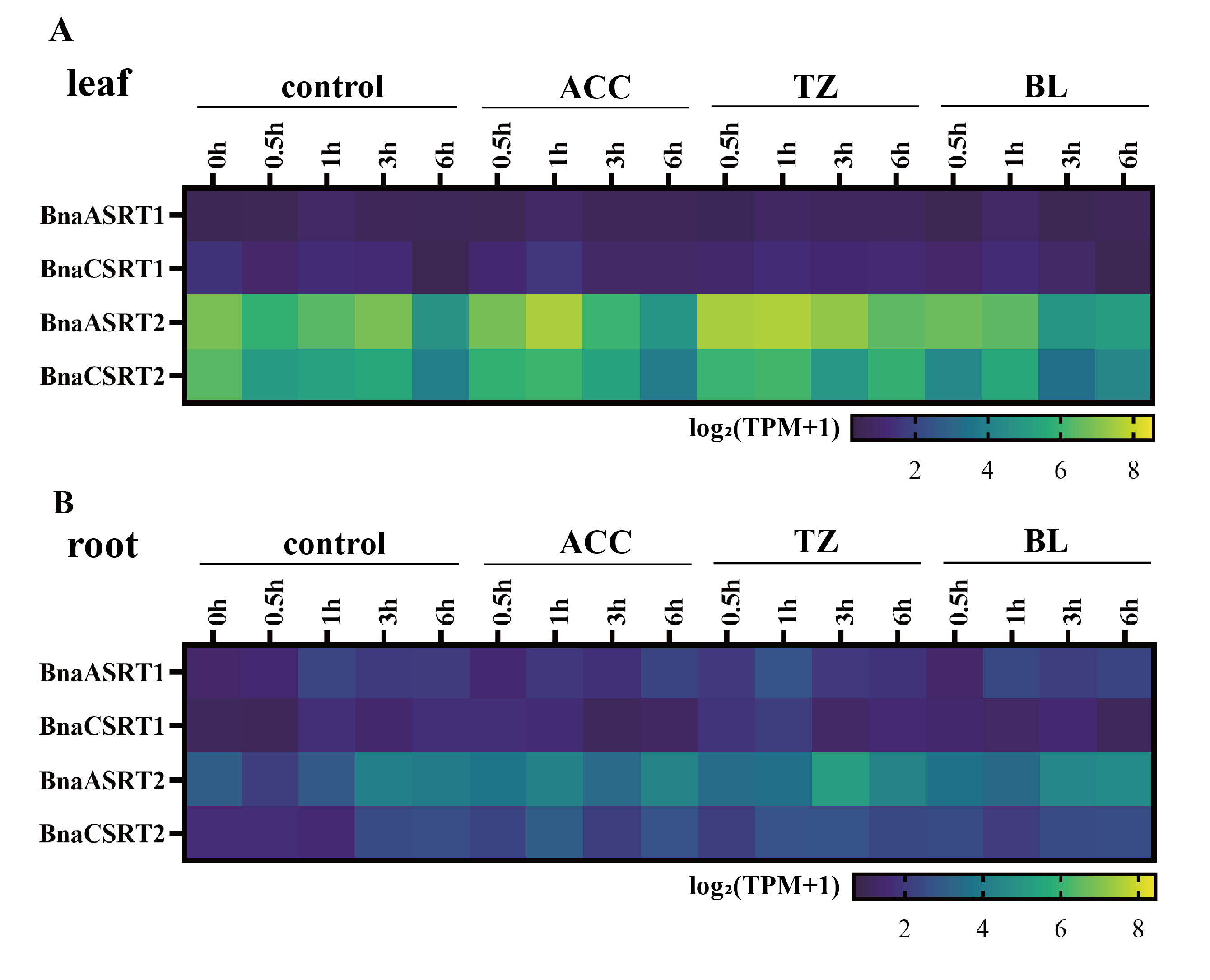

Supplement: S10 Fig — These expression profiles pertain respectively to the leaves (A) and the roots (B) at different time points in response to different hormone treatment (ACC, TZ and BL). (TIF) [file pone.0340688.s011.tif]
